# Research Exploring Physical Activity in Care Homes (REACH): study protocol for a randomised controlled trial

**DOI:** 10.1186/s13063-017-1921-8

**Published:** 2017-04-19

**Authors:** Anne Forster, Jennifer Airlie, Karen Birch, Robert Cicero, Bonnie Cundill, Alison Ellwood, Mary Godfrey, Liz Graham, John Green, Claire Hulme, Rebecca Lawton, Vicki McLellan, Nicola McMaster, Amanda Farrin, on behalf of the REACH Programme Team, Anne Forster, Karen Birch, David R. Ellard, Amanda Farrin, Joan Firth, Beverley Gallagher, Mary Godfrey, Liz Graham, John Green, Rebecca Hawkins, Claire Hulme, Rebecca Lawton, Najma Siddiqi, John Young

**Affiliations:** 1 Academic Unit of Elderly Care and Rehabilitation, Bradford Teaching Hospitals NHS Foundation Trust and University of Leeds, Bradford, UK; 2 School of Biomedical Sciences, University of Leeds, Leeds, UK; 3 Clinical Trials Research Unit, University of Leeds, Leeds, UK; 4 Academic Unit of Elderly Care and Rehabilitation, Bradford Teaching Hospitals NHS Foundation Trust, Bradford, UK; 5 Academic Unit of Health Economics, Leeds Institute of Health Sciences, University of Leeds, Leeds, UK; 6 School of Psychology, University of Leeds, Leeds, UK

**Keywords:** Care homes, Elderly, Feasibility, Cluster randomised controlled trial, Physical activity, Movement, Sedentary

## Abstract

**Background:**

As life expectancy increases and the number of older people, particularly those aged 85 years and over, expands there is an increase in demand for long-term care. A large proportion of people in a care home setting spend most of their time sedentary, and this is one of the leading preventable causes of death. Encouraging residents to engage in more physical activity could deliver benefits in terms of physical and psychological health, and quality of life. This study is the final stage of a programme of research to develop and preliminarily test an evidence-based intervention designed to enhance opportunities for movement amongst care home residents, thereby increasing levels of physical activity.

**Methods/design:**

This is a cluster randomised feasibility trial, aiming to recruit at least 8–12 residents at each of 12 residential care homes across Yorkshire, UK. Care homes will be randomly allocated on a 1:1 basis to receive either the intervention alongside usual care, or to continue to provide usual care alone. Assessment will be undertaken with participating residents at baseline (prior to care home randomisation) and at 3, 6, and 9 months post-randomisation. Data relating to changes in physical activity, physical function, level of cognitive impairment, mood, perceived health and wellbeing, and quality of life will be collected. Data at the level of the home will also be collected and will include staff experience of care, and changes in the numbers and types of adverse events residents experience (for example, hospital admissions, falls). Details of National Health Service (NHS) usage will be collected to inform the economic analysis. An embedded process evaluation will obtain information to test out the theory of change underpinning the intervention and its acceptability to staff and residents.

**Discussion:**

This feasibility trial with embedded process evaluation and collection of health economic data will allow us to undertake detailed feasibility work to inform a future large-scale trial. It will provide valuable information to inform research procedures in this important but challenging area.

**Trial registration:**

ISRCTN registry, ISRCTN16076575. Registered on 25 June 2015.

**Electronic supplementary material:**

The online version of this article (doi:10.1186/s13063-017-1921-8) contains supplementary material, which is available to authorized users.

## Background

Life expectancy has increased dramatically over the last century. While policy and service developments have emphasised alternatives to long-term care, evidence suggests that around one in four older people will spend time in a care home in their last year of life [1] and that the need for such care will persist [2]. In 2011, more than a quarter of a million (291,000) people aged 65 years and over were living in care homes in England and Wales, 3.2% of this population [3].

Research over decades reports that care home residents spend the majority of their time inactive [4]. Lack of engagement in physical activity (PA), defined as “any bodily movement produced by skeletal muscles that results in energy expenditure” [5], has detrimental effects on physical and psychological health and quality of life, and contributes to social isolation [6]. Additionally, an observational study suggests that 97% of residents’ days are spent sedentary (e.g. sitting, watching television), with low levels of interaction with staff and each other [4]. Sedentary behaviour, defined as “any waking behaviour characterised by an energy expenditure ≤1.5 metabolic equivalents (METs) while in a sitting or reclining posture” [7], has been seen to negatively impact morbidity and mortality independently of PA [8]. Consequently, the National Institute for Health and Care Excellence (NICE) [9] issued a quality standard call for “older people in care homes to be offered opportunities during their day to participate in meaningful activity that promotes their health and mental wellbeing”. Meaningful activity can range from activities such as caring for plants and helping around the home, to leisure pursuits such as playing cards.

Existing research has primarily involved the delivery of time-limited interventions by staff external to the care homes [10]. It seems necessary that, if such interventions are to be successfully and sustainably delivered, they need to be embedded in routine practice and that care home staff should be involved in developing and delivering the necessary change [11]. Further, there is increasing emphasis on programmes which reduce the overall time spent sedentary [12] and that do not simply involve short bursts of formally organised PA such as exercise classes. Together, these reinforce the need for action to increase levels of PA in care homes, reduce time spent sedentary, and incorporate greater engagement of care home staff in developing and delivering whole practice change which is embedded in daily life routines.

Through a series of studies we have collaborated with care home staff and residents to develop a multi-component intervention aimed at changing how the routine work of care staff is carried out to encourage and support residents to engage in more PA (above and beyond organised exercise sessions) and reduce the time they spend sedentary. Increasing resident PA and reducing the time they spend sedentary will, we hypothesise, improve residents’ physical and psychological outcomes.

The first study for the Research Exploring Physical Activity in Care Homes (REACH) programme involved ethnographic fieldwork in four care homes to understand the contextual and organisational factors that shape care practice, residents’ existing patterns of PA, and the potential for change. It emerged in this first study, and was reinforced in later studies, that ‘physical activity’ was a difficult concept for care home staff to understand and engage with, particularly in view of the frailty of many residents. Staff linked the notion of PA with specific time-limited activities which might be undertaken by an activity co-ordinator. Our intent was to engage all staff in encouraging residents to undertake small changes in their daily life. The notion of increasing movement and ‘moving more’ had greater resonance and intuitive understanding for staff and residents. Thus, this is the language we adopted in the care home setting. We anticipate that ‘moving more’ will increase levels of PA.

The second study explored the acceptability, validity, and reliability of different measures of PA and sedentary behaviour, which included the development and testing of protocols for the use of accelerometers by residents.

In the third study, the researchers worked alongside a reference group of care home managers, staff, residents, and their relatives/friends to identify barriers and opportunities for change through the process of intervention mapping [13].

In the fourth study, through an action research approach with care home staff and residents in a further four homes, we developed and tested elements of an intervention and change process to reduce residents’ sedentary time and encourage them to ‘move more’. An iterative approach, drawing on the previous work and the implementation literature, was utilised during this process. A novel feature of the work was the involvement of an artist to capture and illustrate pictorially the voice of residents, which resonated with staff in a way that words did not.

Here, we report the protocol for the fifth study, designed to explore the feasibility of training staff to incorporate the intervention into their care home, and the feasibility of trial processes (recruitment, follow-up, data collection) to inform the feasibility and design of a future definitive randomised controlled trial (RCT).

## Methods/design

### Design

The REACH feasibility trial is a pragmatic, multicentre, cluster-randomised controlled trial to explore the practicality and acceptability of implementing a large-scale definitive cluster RCT comparing the REACH intervention plus usual care versus usual care practice alone among permanent residents living in residential care homes in the UK. The REACH intervention is a whole-home intervention designed to assist care home staff to make step-by-step changes in their approach to working with residents, and therefore the care home is the unit of randomisation.

**Fig. 1:**
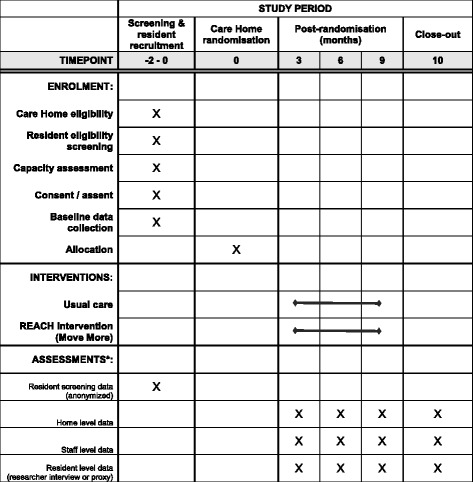
SPIRIT figure

Figure 1 illustrates the timing of all trial processes (Additional file 1).

### Objectives

The study objectives relate to the feasibility trial and the linked process evaluation. The former will involve gathering data around recruitment and follow-up, intervention delivery, assessment of outcome measures, and statistical aspects to inform the feasibility and design of a definitive RCT. The process evaluation, drawing on Medical Research Council guidance on process evaluations [14], will collect data relating to the ‘how’ of the intervention and implementation process (the ‘theory of change’ or assumptions underpinning the programme); the process and quality of implementation as it evolves in real time [15]; and how implementation impacts on, and is shaped by, the organisational and interactional environment of the care home [16].

In summary, the main objectives are to:

1. Explore the optimum strategies to facilitate recruitment, and estimate recruitment and follow-up rates at both care home and resident levels;
2. Assess the compliance and feasibility of wearing an accelerometer to obtain data to measure the level of a resident’s PA (anticipated primary outcome for the definitive RCT) and time spent sedentary;
3. Evaluate the most appropriate outcome measures by assessing the feasibility of obtaining questionnaire data among residents related to physical function, ability and mobility, and psychological well-being;
4. Determine the optimum strategy for collecting data relating to health care resource use and adverse events. The reliability of routinely collected data at both the care home and resident level will also be considered;
5. Assess intervention fidelity in terms of exposure, reach, and quality of implementation, as well as the acceptability to staff and their understanding of the intervention;
6. Explore factors contributing to and hindering organisational and practice change, and the contextual factors that affect variation in the outcomes achieved;
7. Investigate the characteristics of usual care within residential care homes;
8. Provide a preliminary estimate of the effectiveness of the intervention as measured by the volume and level of PA undertaken and the time spent sedentary from the accelerometer data;
9. Calculate reliable estimates to inform the sample size calculations for a definitive trial by assessing the variability in study outcomes in both arms, estimating the intra-cluster correlation coefficient (ICC), and estimating likely cluster size and corresponding between-cluster variability;
10. Investigate the within-trial and long-term incremental cost effectiveness of the intervention compared with usual care.

### Recruitment setting and participants

The study will be conducted in 12 residential care homes in the counties of West and North Yorkshire in the UK. A care home will be considered eligible if:

1. Initial scoping indicates that there are sufficient numbers of permanent eligible residents to enable 8–12 permanent residents to be recruited from that home (homes will later be excluded if researchers find that they are unable to obtain consent and baseline data for at least 5 residents); and
2. There is a manager or nominated person who agrees to sign up to the trial protocol as research lead for the duration of the project and to release staff time for data collection, including supporting the use of the accelerometers and, where appropriate, intervention implementation. Signed agreement is required from the care home manager and care home owner or representative.

A care home will be excluded if:

1. In the view of the research team it is not suitable for inclusion due to being subject to Care Quality Commission (CQC) enforcement notices, admission bans, or relevant moderate or major CQC compliance breaches;
2. It is receiving other special support for specific quality concerns, such as being currently subject to, or have pending, any serious safeguarding investigations, or receiving voluntary or compulsory admissions bans, or is in receipt of local commissioning special support due to quality concerns;
3. It has taken part in any of the earlier studies within the REACH programme;
4. It is taking part, has recently taken part, or is planning to take part, in another trial or initiative that conflicts with the REACH intervention or with the data collection during the course of trial involvement.

All residents in a care home will be exposed to the intervention should the home be allocated to the REACH intervention. Those considered eligible for individual outcome assessment will be permanent residents aged 65 years or over, not terminally ill nor permanently bedbound, and appropriately consented. A permanent resident is defined as residing in the care home and not in receipt of respite, day-care, or short-term rehabilitation.

### Care home recruitment and consent

All residential care homes in a pre-specified catchment area of West Yorkshire will be identified via the CQC website and assessed for eligibility, as far as is possible, via publicly available information. Those deemed potentially eligible at this stage will receive an information sheet via the post with a reply slip to register interest in participation (a copy of this can be found in Additional file 2). Researchers will contact care homes to answer any queries and determine their interest if a reply slip is not returned within 2 weeks. For interested care homes, a researcher from the Academic Unit of Elderly Care and Rehabilitation (AUECR) will complete an initial eligibility assessment via telephone ahead of visiting the care home to determine full eligibility, obtain agreement to participate, and complete the recruitment process. Documented reasons for ineligibility and declining participation will be closely monitored by the trial team at each stage. The target is for a minimum of two care homes to be recruited per month.

To ensure the sample of 12 care homes is reached, in close liaison with Clinical Research Network staff, the Enabling Research in Care Homes (ENRICH; http://enrich.nihr.ac.uk/) network will be contacted to assist with identifying homes in an additional catchment area. All care homes registered on the ENRICH network in the York and Harrogate areas of North Yorkshire will be sent the information sheet via email with contact details to register interest in participation. Colleagues from the local Clinical Research Network will follow-up contact with care homes if no response has been received within 2 weeks. For interested care homes, eligibility will be assessed by the study researcher via telephone and visit as outlined above.

### Resident recruitment and consent

Following care home agreement to participate, all residents will be screened for eligibility. Demographic data will be recorded anonymously by the researcher through discussions with the care home manager and staff members who know the residents well. Residents will not be identified to the researcher by name, and any information provided will be differentiated only by a screening number.

**Fig. 2:**
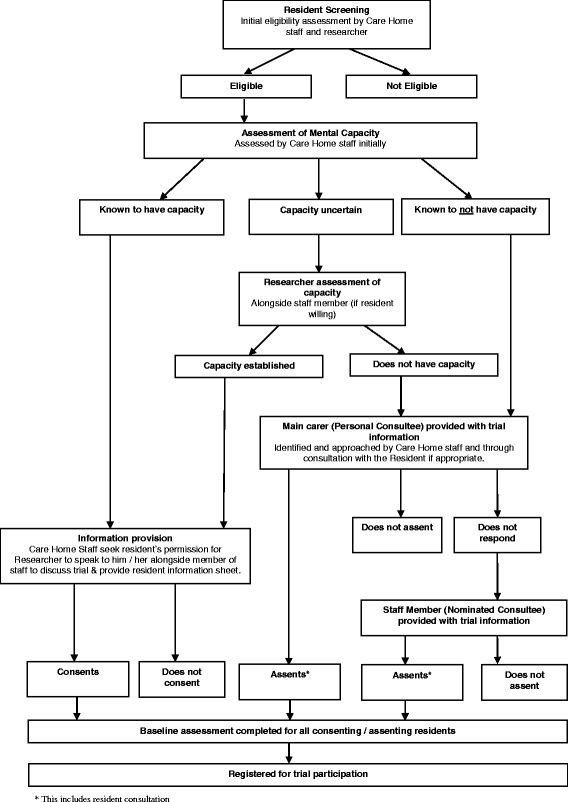
Process for assessing capacity and obtaining consent

An initial assessment of the capacity of each eligible resident to consent will be undertaken by the manager or nominated deputy. All residents will be assumed to have capacity unless assessed to lack capacity in accordance with the Mental Capacity Act (MCA) 2005 guidance [17]. Those with capacity will be provided with information about the study by the researcher and offered at least 24 h to consider participation if they wish. Formal written consent to take part will be sought by the researcher, and ongoing informal capacity assessments conducted by the researcher at each follow-up visit. If a resident’s capacity is uncertain after initial assessment, the researcher will be asked to assist in determining capacity alongside a member of staff if the resident is willing (Fig. 2).

#### Recruitment of those without capacity

It is anticipated that many of the residents will lack the capacity to consent. The MCA requires that those lacking capacity are only included in research that is likely to be of direct benefit to those taking part or to benefit the particular population under study. In this trial, the intervention is delivered at the care home level, hence all residents may benefit directly from enhanced routine PA and indirectly through increasing social engagement. Excluding those without capacity would compromise the generalisability of findings by recruitment of an unrepresentative study sample and excluding this vulnerable population from the benefits of research evidence in improving practice.

Research Ethics Committee (REC) approval includes agreement to involve residents lacking capacity and the process to include them, namely to seek personal consultee or nominated consultee agreement for such residents to participate. For those residents who lose capacity during study participation following the provision of informed consent, an identified consultee will provide advice on a resident’s continued involvement in the trial. Reasons for non-consent, ineligibility, or declining participation either by the resident themselves or their consultee will be closely monitored by the trial management team as part of a regular review of the recruitment process.

All resident and consultee information and consent documentation can be found in Additional file 2.

### Care home randomisation

Care homes will be randomised on a 1:1 basis to the REACH intervention plus usual care or to usual care alone. Due to the small numbers of care homes (trial clusters), stratified randomisation will be implemented to ensure balance between the arms with respect to size of the care home (small/medium ≤40 beds, large >40 beds) and whether or not an activity co-ordinator is in post (characteristics expected to be correlated with intervention implementation and outcome evaluation).

The allocation will be performed centrally by the statistician at the Clinical Trials Research Unit (CTRU) at the University of Leeds using a computer-generated minimisation program with a random element, following recruitment of residents and completion of their baseline assessments. CTRU will provide the random allocation to researchers involved in intervention implementation and adherence monitoring, so that all care home managers can be informed of their care home’s allocation, and arrangements can be put in place for those homes randomised to intervention delivery. Researchers involved in the collection of outcome measures will not be informed of allocation, and efforts will be made to ensure that they remain blind. This will include maintaining separate office locations for ‘blinded’ and ‘unblinded’ researchers and requesting that care homes do not disclose their allocation to these researchers.

Due to the concern that smaller homes may not have enough residents providing baseline data to ensure sufficient outcome data for evaluation (despite initial assessment that there should be 8–12 eligible residents at the care home), homes will only be randomised if there are at least five participating residents providing baseline data.

### Intervention and implementation process

The REACH intervention is designed to be implemented and embedded in routine care delivery within the care home. It has been cumulatively built up from our earlier work and aims to achieve change in how all members of staff work with residents to encourage and support them to engage in more PA and spend less time sedentary. This will utilise the terminology ‘moving more’. The intervention will focus on change in four domains of daily routines that embrace: independent/supervised movement of residents to get about; incorporating movement in social and leisure activities; providing opportunities for residents to engage in ‘meaningful’ activities; and encouraging residents to do as much of their own self-care and instrumental activities of daily living as is possible for them.

Implementation will comprise a cyclical service improvement approach. It will be led by a team involving key stakeholders in the change: staff, residents (if possible), and their relatives/friends facilitated by a senior member of care home staff. This synthesises a top-down and bottom-up approach to leading change [18]; this means that the active involvement of senior staff with the authority and legitimacy to drive the change process forward is aimed at securing organisational commitment to introducing and embedding the programme (top-down), and the engagement of those directly involved in action to deliver it ensures that their views and experiences will inform the pace and direction of change (bottom-up) [19, 20].

The intervention and implementation process does not prescribe particular kinds of changes that staff should make to increase residents’ movement, but remains flexible to be adapted to each care home’s needs.

Several strategies are in place to support implementation: identification of an intervention lead and core team in each home; provision of a manual, including an ‘Ideas Bank’ of resources to assist staff in getting started and keeping going; and training and support comprising a series of three interactive workshops provided individually to each home.

We have been guided by the template for intervention description and replication (TIDieR checklist [21]) and the treatment fidelity framework provided by the National Institutes of Health’s Behavioral Change Consortium [22] to capture the intervention (its rationale, materials, procedures, etc.) and to guide approaches to implementation and fidelity monitoring.

### Usual care

Usual care is defined as ‘normal’ care delivered within the care home and will continue in both arms. No restrictions will be imposed on current practices or on care homes undertaking additional training and development as part of usual care. Details of general day-to-day activities will be recorded via researcher observations within public areas of a care home and completion of a pro-forma developed for the purposes of this study.

### Trial data collection

The intention is that the intervention becomes embedded within the care home (if so randomised); thus it is important to assess the impact of the intervention on the care home as a whole. Consequently, data will be collected at the level of the care home (including staff) as well as from individual consenting residents at baseline (prior to randomisation) and at 3, 6, and 9 months post-randomisation. Data will be collected by trial researchers who will visit each participating care home at each follow-up time point. Personal information will be held centrally, in accordance with consent, by the research team to facilitate follow-up contact, but will be stored and processed separately to all other data collected for the purposes of the trial. In order to assess level of blinding, all outcome assessors will be asked to report immediately if they are unblinded.

A summary of all assessments to be used is provided in Table 1.

**Table 1:** Summary and timing of assessments

Assessment	Method of completion	Timeline				
		Screening	Baseline	3 months	6 months	9 months
Care home eligibility	Researcher assessment	X				
Resident screening (demographics)	Researcher assessment	X				
Physical Activity and Mobility in Residential Care Scale (PAM-RC) and Barthel index	Researcher interview/self-completion (S)	X		X	X	X
Resident consent (including consultee)	Self-completion (R) (witnessed)	X				
Resident eligibility	Researcher assessment	X				
Contact details (including consultee, staff informant/proxy if applicable)	Researcher assessment	X				
Care home demographics	Researcher interview (S)		X	X	X	X
Staff profile	Researcher interview (S)		X	X	X	X
Home level mortality rates, hospital admissions, HCP contacts, and adverse events	Researcher interview (S)		X	X	X	X
Staff details questionnaire (including the Person-centred Care Assessment Tool (P-CAT))	Self-completed (S)		X	X	X	X
Functional Ambulation Classification (FAC), and the Elderly Mobility Scale (EMS)	Researcher interview/self-completion (S)		X	X	X	X
Level of cognitive impairment (6-CIT)	Researcher interview (R)		X	X	X	X
Mood (Geriatric Depression Scale (GDS))	Researcher interview (R)		X	X	X	X
Perceived health (EQ-5D-5 L)	Researcher interview/proxy completion (R/SP)		X	X	X	X
Quality of life (DEMQOL)	Researcher interview/proxy completion (R/SP)		X	X	X	X
Accelerometer measurements	Researcher assessment		X	X	X	X
Health Economics Questionnaire	Researcher interview (S)/review of care notes		X	X	X	X
Service usage	Routine data sources		Collected throughout			
Hospital admissions/safety reporting	Researcher assessment/routine data sources		Collected throughout			
Usual care review	Researcher assessment (observations)		X	X	X	X
Intervention delivery and adherence	Researcher assessment		X	X	X	X

*6-CIT* Six-item Cognitive Impairment Test, *DEMQOL* Dementia quality of life tool, *EQ-5D* EuroQoL five dimensions, *HCP*, Health Care Professional *R* resident, *S* care home staff, *SP* staff proxy

#### Care home-level data

The care home manager/nominated staff member will be asked to complete a care home booklet to provide information on the staff and resident profile of the home, and anonymous home-level data relating to hospital admissions, general practitioner (GP) call-outs, mortality rates, and falls in the last 3 months. At baseline, as part of the screening process, anonymous Physical Activity and Mobility in Residential Care Scale (PAM-RC) (Whitney et al. manuscript submitted) and Barthel Index [23] data will also be collected for all residents to establish the profile of PA, participation in activities of daily living/self-care at the level of the care home, and ambulatory capacity.

#### Staff data

At each data collection time point all staff who have face-to-face contact with residents, with the exception of those acting as a nominated consultee, will be asked to provide basic demographic data about themselves and complete a questionnaire regarding their experience of person-centred care provided in the care home (Person-centred Care Assessment Tool (P-CAT)) [24].

#### Resident-level data

Participating residents will be asked to complete questionnaires (via researcher interview) assessing their level of cognitive impairment (six-item Cognitive Impairment Test (6-CIT)) [25], mood (Geriatric Depression Scale (GDS)) [26], perceived health (EuroQol five dimensions (EQ-5D-5 L)) [27], and quality of life (dementia quality of life tool (DEMQoL)) [28]. Proxy completion of the EQ-5D questionnaire by a member of staff who knows the resident well will also be undertaken for all participating residents. Additionally, where residents are unable to answer the questions themselves, proxy completion of the DEMQoL questionnaire will be undertaken. Appropriate staff members will also be asked to complete questionnaires about physical function and mobility (Functional Ambulation Classification (FAC) and Elderly Mobility Scale (EMS])) of resident(s) they know well. Where possible, the same staff member will provide data for a particular resident throughout the study. Any staff member acting as a nominated consultee will not act as a staff proxy informant so that they remain independent of the research.

Residents who give their consent will be asked to wear an ActiGraph wGT3X-BT accelerometer (Actigraph, Pensacola, FL, USA) on the right hip, secured using an elasticated belt, during waking hours over the course of 7 days to record PA through movement counts collected over 60-s epochs. Valid wear time will be defined as ≥8 h 25 min on ≥4 days. For residents who provide valid data, the amount of time spent in various PA categories [29] and in sedentary behaviour will be identified using cut-points applied to the vertical axis accelerometer counts per minute (cpm) (see Table 2). A detailed protocol will be followed for administration of the accelerometers which will take place after the data collection defined above to ensure that accelerometer wear does not interfere with residents’ routine PA levels and influence their questionnaire outcome assessments.

**Table 2:** Accelerometer cut-points

Accelerometer counts per minute (cpm)	PA classification	Examples of activities
<100 cpm	Sedentary	Sitting, reclining
100–759 cpm	Low intensity PA	Upper body movements
760–2019 cpm	Light intensity PA	Self care, slow walk
≥2020 cpm	Moderate-vigorous (MV) PA	Walking, sit-stand transfers

*PA* physical activity

Adverse event data will be collected by a researcher on a monthly basis and the best method of collecting service use data will be explored, including data routinely recorded by the care home as well as, where possible, via receipt of hospital attendance data from NHS Digital and relevant Acute Hospital Trusts.

#### Process evaluation

A mixed-method approach will be employed with different stakeholders, at different levels (all homes; all intervention homes; purposive sub-sample of intervention and control homes).

Data will be collected across all 12 trial homes to describe usual care and the contextual factors within and outwith the care home environment that affect resident PA and sedentary time over the period of the trial. Researchers will use a qualitative, trial-specific observational tool at each data collection time point to obtain insights into residents’ PA at different times, in different locations, and in respect of residents with different types of need. They will also complete a chronology of organisational (including staffing) policy and practice changes that have occurred in the 3 months preceding data collection. Qualitative interviews will be conducted with a senior member of staff in each home at the conclusion of the trial by the dedicated process evaluation researcher(s) to explore the value attached to ‘moving more’ and how this may have changed over the period of the research.

Across all intervention homes, data will be collected on the intervention and implementation process by the process evaluation researcher and programme facilitators. This will include audio recordings of the workshops and collection of documentary data relating to the cyclical process of change over time (observation, action planning, and review sheets). Information will be gathered on which residents have been chosen for action and their characteristics; which domains of “moving more” have been targeted and with what effect; and which staff, occupying which roles, are nominated to provide support and encouragement to residents to engage in more PA. Qualitative interviews will be undertaken with the care home’s intervention lead to explore how the intervention is understood, engaged with, and enacted by different stakeholders in the real life context of individual care homes, and examine how both intervention and contextual factors facilitate, delay, or impede change.

Within a sub-sample of two intervention and two control homes, ethnographic observations of levels of movement within public areas will be conducted by the process evaluation researcher(s) alongside informal discussion with residents about their experience of, and attitudes to, ‘moving more’, and interviews with staff generally about the value they attach to PA and practices in relation to it. These data will allow in-depth evaluation of how the intervention enhances attitudes towards and practices relating to PA among staff and residents over the study duration, over and above any changes that may be introduced within the control homes. Within the intervention homes, it will also contribute to understanding whether change occurs in the pattern of PA and sedentary behaviour over time, as well as in the engagement and reach of the intervention to staff and residents.

Within the sub-sample of two intervention homes, data on the theories of change and how these are enacted and impact on the pattern of movement over time will be collected through observation of implementation team meetings, review of documents relating to the service improvement cycle, and interviews with members of care staff, including the intervention lead, over the course of implementation. Since implementation is conceived of as a process and not a one-off event [30], documenting the process as it evolves over time and in relation to contextual factors in the care home is considered necessary. Analysis will be based on the principles of grounded theory [31].

#### Economic evaluation

A within-trial cost effectiveness analysis will be undertaken from the perspective of the NHS and Personal Social Care Sectors. It will compare costs and outcomes over the 9-month follow-up period between each arm. The analysis will use quality-adjusted life years (QALYs) derived from responses to the EQ-5D-5 L and EQ-5D-5 L proxy [32]. Cost estimates will be based on NHS resource use (accident and emergency/unplanned hospital admissions, falls, soft tissue injuries, NHS and non-NHS service use) collected from care notes and via a health economics questionnaire completed by researcher interviews with staff at 3, 6, and 9 months post-randomisation. The resources associated with implementation and delivery of the intervention will be included in the analysis. These will be based on routine data such as administrative records, trial-specific data collected during implementation, and a detailed description of the implementation and development process provided by the researchers involved in intervention implementation. Unit costs for health service resources will be obtained from national sources such as the Personal Social Services Research Unit (PSSRU), the British National Formulary (BNF), and NHS Reference cost database [33]. The non-parametric bootstrap method will be used to produce a within-trial probabilistic sensitivity analysis of the incremental cost effectiveness ratio. Longer term cost effectiveness will be estimated using a decision analytical model. The long-term cost effectiveness modelling will adopt the same perspective as the within-trial analysis and will follow good practice guidelines [34]. A value of information analysis will be undertaken [35].

### Sample size

We aim to recruit 12 residential care homes (six per arm) with an average of 8–12 participating residents per home, based on recruitment in our earlier studies. A formal power calculation is not appropriate in this feasibility trial as effectiveness is not being evaluated. However, this target sample size allows us to maintain sufficient power for an effect size of 0.50 and above across the outcome measures while increasing the Type I error rate from 0.05 to 0.20 (Table 3). Increasing the Type I error rate provides assurance that the intervention is promising and warrants further evaluation given that we are making a preliminary and non-definitive randomised comparison of the intervention with usual care. It is recognised that, with an increased false-positive rate in this feasibility trial, a definitive trial may not show an intervention effect at the care home level; however, sufficient changes at an individual level may be detected which would also be of interest. The results generated from this study will be used to inform the sample size for a definitive trial.

**Table 3:** Anticipated power for a range of effect sizes across outcome measures with six clusters per arm, not accounting for losses to follow-up

Effect size	ICC	Power (cluster size = 8)	Power (cluster size = 12)
0.40	0.05	65%	74%
	0.10	59%	65%
0.50	0.05	79%	87%
	0.10	72%	78%
0.60	0.05	89%	94%
	0.10	83%	88%

*ICC* intra-class correlation coefficient (which represents the amount of correlation between observations (residents) within a cluster (care home))

### Analysis

Statistical analysis of the quantitative elements of the trial is the responsibility of the CTRU statistician, and a detailed statistical analysis plan will be written and agreed before data are analysed. No formal interim analyses are planned and final analysis will take place when all available data have been received and the database has been cleaned and locked. Analyses and data summaries will be conducted on an intention-to-treat population, defined as according to randomisation and regardless of non-compliance with the protocol or withdrawal from the study. The focus of analyses will be descriptive statistics and confidence interval (CI) estimation rather than formal hypothesis testing, with the exception of a preliminary estimate of effectiveness.

#### Recruitment uptake and follow-up

To assess feasibility of recruitment, the number of homes and residents screened, eligible, and providing consent/assent will be summarised, alongside reasons for non-participation. Retention during follow-up, including the number, timing, and reasons of withdrawals, will be reported overall and by study arm to assess whether there are any systematic differences between the arms which could be attributed to the intervention. The number of residents moving in and out of the care home will also be recorded.

#### Intervention delivery and compliance

Actual intervention delivery will be assessed using trial-specific documentation (case report forms), as well as through detailed process evaluation work. Documentation will allow reporting of the number of care homes failing to progress through implementation milestones and reasons for failure to implement any aspects of the intervention. This includes: the intervention workshops (including a summary of workshop content and attendees); the presence and content of action plans; and the level of support provided by the research team members responsible for intervention training to enhance intervention implementation. Implementation processes will be assessed in more depth via qualitative analysis of the process evaluation data, including observations of implementation team meetings, interviews with staff, informant conversations, and detailed field notes.

#### Characterising usual care

Specific initiatives relating to enhancing any part of resident care and any materials displayed in the care homes relating to PA (or the context of ‘moving more’), such as leaflets or posters, will be summarised at each time point and by arm. Care home and staff characteristics will be summarised to inform the context in which the intervention was delivered, as well as staff turn-over resulting in employment at other trial care homes to assess the degree of contamination.

#### Assessment of outcome measures

The number and proportion of questionnaires received at each time point will be summarised overall and by study arm. Point estimates for resident scores will be calculated for each outcome measure, together with CIs for the differences in scores between the study arms. The amount of missing data will also be presented.

To inform the feasibility of monitoring levels of PA and sedentary behaviour, the number and proportion of residents who wear an accelerometer and the daily wear time will be summarised by arm at each time point, alongside the reasons for residents not wearing the accelerometer.

#### Preliminary estimate of the effectiveness of the intervention

Through the use of accelerometers we are able to derive several different PA and sedentary behaviour end-points. We will be able to assess the amount of time residents spend sedentary and in low, light, and moderate to vigorous intensity physical activity (MVPA). We can also assess the number of daily bouts (defined as consecutive minutes) of specified lengths spent in these categories. Examples of such end-points include:

- Percentage of time spent sedentary and in low-intensity categories
- Number of daily sedentary bouts of >60 min
- Number of daily sedentary bouts of >30 min
- Number of daily bouts of low intensity >5 min
- Total length of time spent sedentary or in low-intensity physical activity
- The total time spent achieving any MVPA

For each PA outcome of interest (as detailed above) measured using the accelerometers, point estimates will be calculated by study arm based on cluster-level summaries, together with a range of CIs (95%, 80%, 67%, and 51%) to give a greater indication of the direction and effect size we might expect to see in a large-scale trial. Data will be reported separately at baseline and at each follow-up time point.

Following a review of the summaries for each of the PA outcomes, the most appropriate end-point for future use in the large-scale trial will be selected. This decision will be made on the basis of both the summary data and any emerging evidence on physiological effects (for example, that increasing the number of breaks in sedentary behaviour is more important than reducing the total amount of sedentary behaviour). No information will be provided split by treatment arm at this stage to avoid selection bias. Once this decision has been made we will then summarise these end-points by trial arm.

A preliminary estimation of effectiveness will be carried out using methods appropriate for cluster randomised trials with a small number of clusters [36]. Point estimates of the 9-month outcome in each arm will be used to obtain difference or ratio estimates of the intervention effect depending on the type of outcome chosen. If the distribution of the summary measures in each arm is skewed, a logarithmic transformation will be considered. Cluster-level summaries will be used to obtain an 80% CI for the intervention effect and hypothesis testing will be conducted at the 20% significance level using the *t* test.

Adjustment for covariates will be carried out using a two-stage process. In the first stage, a standard regression model including the covariates of interest, but excluding the intervention effect, will be fitted to calculate cluster-specific expected values. Expected and observed values will be compared by computing a residual for each cluster. These cluster residuals will then be analysed using methods based on the *t* test in the second stage of analysis.

To inform the sample size for a definitive trial, unadjusted and adjusted estimates of the ICC will be calculated alongside the amount of between-cluster variability in study outcomes and cluster size.

#### Process evaluation

Qualitative data (field notes, interviews, informant conversations, audio workshop recordings) will be analysed using grounded theory analytic strategies [31] combining simultaneous data collection and analysis, constant comparison, and search for negative cases. This approach provides a more robust, systematic, and in-depth approach to addressing issues of context and process over time, critical in this study.

Analysis will be conducted at several levels. For each care home, multiple sources of data will be drawn upon to provide a descriptive narrative account of the physical environment, organisation, staffing, resident profile, and pattern of PA and sedentary behaviour of residents. For intervention homes the process of understanding, engaging with, and implementing the intervention in the context of each care home will be analysed, drawing on documents and interviews with the intervention leads. Through comparison between intervention homes we will identify how the level of engagement with the intervention, the domains of action and the reach of the intervention across staff and residents vary, the factors that shape these, and how they affect the outcomes of the trial. Additional data relating to the actual process of implementation in time from the sub-sample of intervention homes will enable exploration of the theory of change underpinning the intervention and implementation process.

#### Progression criteria for continuation to the definitive randomised controlled trial

Guidelines for progression to a definitive RCT are based on a traffic light system of green (proceed to RCT design), amber (review RCT design and/or implementation, then proceed), red (stop and do not proceed), and are detailed in Additional file 3.

### Trial organisation and governance

The REACH trial is sponsored by the Bradford Teaching Hospitals NHS Foundation Trust and is co-ordinated by the Academic Unit of Elderly Care and Rehabilitation (Bradford Teaching Hospitals NHS Foundation Trust and University of Leeds) and the CTRU (Clinical Trials Research Unit, University of Leeds). The trial management group consists of the co-applicants and the teams from the co-ordinating units. The study will be conducted in accordance with the Research Governance Framework for Health and Social Care (2005) and CTRU standard operating procedures.

Overall trial supervision is provided by the Programme Steering Committee (PSC), with an independent Chair and ‘Patient and Public Involvement’ (PPI) representation. A sub-group of the PSC will perform a safety monitoring function since a separate data monitoring and ethics committee is not required for a feasibility trial of this nature and duration.

Data will be entered, managed, and monitored for quality and completeness by the CTRU. Missing data (except individual items collected via questionnaires) will be chased until received, confirmed as not available, or the trial is at analysis. Data will be stored and managed in accordance with the provisions of the Data Protection Act (1998).

### Dissemination

Results of the study will be published in peer-review publications and will be presented at national and international conferences. We will work with the PPI representatives to develop lay reports to disseminate research findings to resident and relative groups and the care home staff at participating homes.

Authorship will be agreed in accordance with the REACH Programme publication policy and in line with International Committee of Medical Journal Editors (ICMJE) recommendations.

## Discussion

As the population ages the numbers at the oldest ages will increase the fastest. In 2008, there were 1.3 million people in the UK aged 85 years and over; this is projected to rise to 1.8 million by 2018 [37]. One consequence is an increase in demand for long-term care which, despite the increased emphasis on community care [38], will remain a necessary component of health and social care provision [2]. Residents of care homes are amongst the frailest in our population with significant health and social care needs [39, 40]. It is reported that older people [41] and care home residents undertake little PA [4, 42] and in fact spend the majority of their time sedentary [43]. Although “there is sufficient evidence to support a recommendation to reduce sedentary behaviour in older adults” [42], the majority of such work relates to younger people, and the implications for older adults are not yet robust. Decreasing levels of PA and increasing dependency have many adverse effects. Reduced PA and mobility problems compound health difficulties by directly affecting physical and psychological health, and reducing opportunities to participate in social activities [6].

We aim to address this challenge in our programme of work in which we are evaluating an intervention which actively engages staff within care homes to embed strategies to increase PA and decrease sedentary behaviour during the daily routines of the care home and its residents. This ‘whole-home’ intervention moves away from a model of time-limited provision of activities such as exercise classes.

Research in this setting in challenging; this feasibility trial will address important methodological issues, assess intervention implementation, and collect data to inform a definitive RCT to evaluate an intervention which addresses a central component of care for frail, elderly people. The work undertaken will also provide guidance and tested protocols for all research undertaken in the care home setting.

### Trial status

The study commenced recruitment of care homes in August 2015, and recruitment of residents in December 2015; 138 residents at 11 care homes had been recruited as of 31 August 2016. The study is projected to complete recruitment by September 2016.

## Additional files

Additional file 1: SPIRIT 2013 checklist: recommended items to address in a clinical trial protocol and related documents. (DOC 123 kb)

Additional file 2: REACH trial information and consent documentation. This document includes all information sheets and consent forms provided to the care home manager, residents, relatives, and staff members involved in the trial. (PDF 1306 kb)

Additional file 3: Progression criteria for continuation to the definitive randomised controlled trial. This document details guidelines for progression to a definitive randomised controlled trial (RCT) which are based on a traffic light system of green (proceed to RCT design), amber (review RCT design and/or implementation, then proceed), or red (stop and do not proceed). (DOCX 20 kb)

## Abbreviations

6-CIT: Six-item Cognitive Impairment Test
AUECR: Academic Unit of Elderly Care and Rehabilitation
BNF: British National Formulary
CI: Confidence interval
CQC: Care Quality Commission
CTRU: Clinical Trials Research Unit
DEMQoL: Dementia quality of life tool
EMS: Elderly Mobility Scale
ENRICH: Enabling Research in Care Homes
EQ-5D: EuroQoL five dimensions
FAC: Functional Ambulation Classification
GDS: Geriatric Depression Scale
GP: General practitioner
ICC: Intra-cluster correlation
MCA: Mental Capacity Act
METS: Metabolic equivalents
MVPA: Moderate-vigorous physical activity
NHS: National Health Service
NICE: National Institute for Health and Care Excellence
NIHR: National Institute for Health Research
PA: Physical activity
PAM-RC: Physical Activity and Mobility in Residential Care Scale
P-CAT: Person-centred Care Assessment Tool
PPI: Patient and Public Involvement
PSC: Programme Steering Committee
PSSRU: Personal Social Services Research Unit
QALY: Quality-adjusted life year
RCT: Randomised controlled trial
REACH: Research Exploring Physical Activity in Care Homes
REC: Research Ethics Committee
