# Supplementary material for: Research Exploring Physical Activity in Care Homes (REACH): study protocol for a randomised controlled trial
Source: Trials. 2017 Apr 19;18:182. doi: 10.1186/s13063-017-1921-8 (PMC5395795; doi:10.1186/s13063-017-1921-8)
Supplement: Supplementary file 2 — REACH trial information and consent documentation. This document includes all information sheets and consent forms provided to the care home manager, residents, relatives, and staff members involved in the trial. (PDF 1306 kb) [file 13063_2017_1921_MOESM2_ESM.pdf]

The REACH Study

Research **E**xploring physical **A**ctivity in **C**are **H**omes

A Summary for Care Homes

Invitation to participate

- We are inviting you to take part in a research project called the REACH study.
- This information sheet gives you details of the study so you can decide whether or not you wish to take part.
- If anything is unclear, or if you would like more information before you feel able to decide then please get in touch with the Researcher (contact details below).

(Please turn over)

How to contact us:

If you have any questions about this study, please contact:

Name of Researcher: <<insert>>
Telephone: <<insert>>

Or please contact the leads for this study:
<<insert>>

This project was funded by the National Institute for Health Research PGfAR (project number RP-RG-1210-12017)

1. Why are we doing this study?

Research shows that increased movement can improve health and wellbeing. We are seeking to develop a whole home intervention which will enhance residents' opportunity for movement, thereby increasing physical activity levels and reducing sedentary behaviour.

Some care homes taking part in this study will use a new approach to increasing movement, whilst others will carry on as normal. We will be able to compare information we collect from each home to help us to see if there are any differences between homes that use the new approach and those that don't.

The study is being carried out by Researchers at Bradford Teaching Hospitals NHS Trust and the University of Leeds. It also forms part of a PhD thesis looking at measuring levels of movement in care homes, which is being undertaken by a Research Assistant who will be working with the care homes.

2. What will happen in the study?

Care Home Selection

We plan to ask 12 care homes across Yorkshire to take part in this research. Care homes will be randomly selected and invited to participate. You have received this information sheet as your care home has been selected in this way.

Confirming Care Home Eligibility

The study Researcher will contact you within the next week to discuss the study in more detail and to see if you are interested. If your care home is interested in taking part, the Researcher will need a bit more information to confirm you are definitely eligible to take part. If you are able to go ahead, the study Researcher will schedule a mutually convenient time to visit your care home to explain the study in full and obtain written agreement from the Care Home Manager (and Organisation if needed) for your care home to take part.

Identifying Residents

Once we start the study, if your home is eligible and you agree to take part, we will ask the Care Home manager (or delegate) to assist the Researcher in assessing residents' eligibility to take part, and in assessing their capacity to consent to take part. Where residents do not have capacity, we would ask the care home to send a study letter to a family member / friend (or approach them in person) to see if they would be willing to provide agreement to participate (assent) on the resident's behalf, in line with the Mental Capacity Act.

This project was funded by the National Institute for Health Research PGfAR (project number RP-RG-1210-12017)

Resident Consent

Before it is decided whether or not a care home will use the new approach, residents will be asked to provide their consent to be involved in this study. We appreciate that there may be some residents who are unable to provide consent themselves but who would still like to take part in the study. In these instances, we will firstly ask the care team to speak with the resident's relative/close friend who may be willing to provide agreement on the resident's behalf. In some cases we may ask a staff member to provide this agreement (if the resident does not have a relative/close friend who is able to provide agreement to take part). If they give consent (or assent) to be involved, this will mean a Researcher from the study will visit to collect some information about their physical activity and ask them some questions on four occasions over 9 months.

Care Home 'Allocation' - Usual Care or 'MoveMore' (the intervention)

Once all residents have had the opportunity to join the study, care homes will be allocated to use the 'MoveMore' intervention alongside 'usual care' or continue with 'usual care' (i.e. carrying on doing what you would normally do). The best way to compare the two approaches is deciding by chance ('randomly') who gets which one - this is called 'randomisation'. So in this study your care home will be either part of the 'usual care' group of homes or the 'MoveMore' group of homes. At the end of the study the two groups can be compared to see whether one approach is more helpful than the other. In this study there is therefore a 50:50 chance of either continuing with usual care, or having usual care plus the 'MoveMore' intervention. Neither the Researchers nor the care home can influence whether a care home is chosen to use 'MoveMore' - this is done randomly by a computer at the Research Office (at the University of Leeds).

The information we collect from both the 'MoveMore' and 'usual care' homes is equally important for the research.

We would compensate you for any time you and your staff spend providing information to the Researcher.

'MoveMore' (the intervention)

'MoveMore' is a package of information, suggestions and practical ideas which can be used to help care homes to introduce more movement into residents' every day care home life. The package is based on a review of what has been tried previously in other studies, through observations within other care homes, and by discussing what might work best with other care home residents and staff.

If you are allocated to use 'MoveMore', members of the research team will arrange three workshop sessions with a group of staff to explain the package and how to

This project was funded by the National Institute for Health Research PGfAR (project number RP-RG-1210-12017)

tailor this to fit in with your home's every day activities. These workshop sessions will be audio recorded; however, staff members have the right to decline this if they wish. After the workshop sessions it would be up to staff to find the most appropriate ways of making changes to increase movement amongst residents. We would ask staff to record what changes were made so we could see what works well and what is less helpful. Homes not randomly allocated to use 'MoveMore' (control group homes) will be offered the chance to train staff to use the new approach at the end of the study, if they wish.

Research visits and assessments

All research needs information (data) to be able to report results at the end of the study. For this study we would ask to visit your care home to collect data from residents about how they are doing, as well as to collect some information from staff and from care home records.

A study Researcher would visit each care home to collect information four times over 9 months. One Researcher would visit the care home for around two to four weeks to collect this information at each time point.

At the first visit we would like to collect some information about each care home (e.g. number of staff and roles) from the Care Home manager or nominated staff member. This would be anonymised and would help us to generally describe the homes involved in the research.

At each visit a researcher will sit for a few short periods of time to observe activities happening in the public areas of the home. These observations will not focus on individual residents but instead will be used to gain an insight into the general day to day activities taking place in the home.

We will invite residents to assist us in the study by wearing activity monitors (a small device very similar to a pedometer) at each of the four Researcher visits. This will enable us to record levels of movement among residents. The activity monitors will be worn round the waist during the day for around 2 to 5 days at each of the four Researcher visits. We will also offer training to all staff members in how to use the activity monitors as we may ask them to assist in putting them on and taking them off; we may also ask for a record to be kept of how long each resident has worn their monitor.

We will also ask residents to fill in some questionnaires with the Researcher at each visit. However, because of communication and memory difficulties not all residents will be able to give us information and we may therefore ask staff members who know the resident(s) well to help with this.

This project was funded by the National Institute for Health Research PGfAR (project number RP-RG-1210-12017)

Residents would be free to stop taking part in one or more aspects of the study at any time.

We will ask staff members to complete a questionnaire about themselves and we will also ask some staff to help by providing information about residents' physical activity and mobility at each Researcher visit, for example whether the resident is able to walk unaided.

The Researcher may have some discussion sessions (focus groups) with a small group of staff and residents to explore what aspects of the study worked well and which aspects could be improved.

3. What do we do if we're interested in taking part?

A Researcher will contact you in the next week to discuss the study in more detail. If you express an interest in taking part the Researcher will ask you some questions to confirm your care home is eligible, and schedule a visit to your care home to discuss the study in more detail.

Please note that by expressing an interest you are not committing your care home to take part. You will receive further information about the study and what participating will entail and will have every opportunity to discuss the study in detail with Researchers.

4. What if we don't want to take part?

If you are not interested in participating please inform the Researcher when they contact you and they will not contact you again.

5. What are the advantages and risks of taking part?

Taking part will help us gain an understanding of how the new approach (MoveMore) works in practice, and help us to see if we can collect the information we are hoping to obtain. This will help us to find out if it would be feasible to run a large scale trial to test if the intervention is effective across many care homes. We would hope that, should we run a large scale study, this may lead to new ways of helping other residents to increase their movement (and possibly improve their health and well-being) in the future. Other Care Homes who have taken part in research have felt that participation was viewed positively by the Care Quality Commission (CQC).

We do not expect there will be any risks in taking part.

6. More information about taking part

Will our taking part be kept confidential?
If you decide to provide data for the REACH study, the information collected about you, your home, your staff and the residents during the course of the study will be kept strictly confidential. The information collected will be recorded on paper forms and sent securely from your care home to the University of Leeds and the Bradford Institute of Health Research. This information will be securely stored at the Research Office at Leeds University on paper and electronically, under the provisions of the 1998 Data Protection Act. At the end of the study, your data will be securely archived for a minimum of 5 years. Arrangements for confidential destruction will then be made.

It is possible that the information you provide for this study may be shared with other research teams to answer new research questions in the future. If this happens the information would be anonymised, meaning your care home name, staff and resident names would never be included, so that no-one would be able to identify you or any study participants from it. Other research teams would not be given any personal details about who participated in the study.

What will happen to the results of the study?
The results of the study will be shared with people living in care homes and their families, care provider organisations and their staff, policy makers, Researchers and the general public. We will share the results through writing articles for magazines and journals, through speaking at conferences and other public events, through producing information leaflets and through the web-pages of the organisations involved. No information that might identify you will ever be included in information we share about the study results.

We will produce a specific information leaflet about the results of the study for staff and residents. These will be sent to each participating care home. If you leave the care home before the study ends then you can ask for us to send you a copy to an address of your choice when the study ends.

Who is organising, funding and reviewing the research?
The study is being organised and supervised by Bradford Teaching Hospitals NHS Foundation Trust and The University of Leeds. It is funded by research funding from the Department of Health.

All research is looked at by an independent group of people called a Research Ethics Committee to protect the safety, rights, wellbeing and dignity of those taking part. This

This project was funded by the National Institute for Health Research PGfAR (project number RP-RG-1210-12017)

study has been reviewed and approved by the East of England – Norfolk Research Ethics Committee (REC Reference: 15/EE/0125).

What will happen if Researchers are concerned an individual may be experiencing abuse?

It is possible that residents may disclose information to the Researcher, or the Researcher may have concerns that the individual may be experiencing abuse, or is at risk of abuse. In such circumstances the Researcher will follow Bradford Teaching Hospital NHS Trusts' Safeguarding Adults policy. The Researcher will discuss their concerns immediately with the Lead Researcher and if they are in agreement, the relevant persons will be contacted as soon as possible, this may be social services, GP, community care team or the acute trust.

Researchers may also observe untoward incidents whilst within a care home. In this occurrence, the Researcher will immediately inform the manager of the care home and will discuss this with the Lead Researcher. If appropriate the NHS Safe Guarding for Adults procedures will be followed.

7. Questions?

If you have any questions or would like more information, you can speak to the Researcher, whose details can be found on page 1 of this information sheet.

If you would like further information about research in general, the UK Clinical Research Collaboration (a partnership of organisations working together on research in the UK) have published a booklet entitled 'Understanding Clinical Trials'. Contact UKCRC: Tel: 0207 670 5452; website www.ukcrc.org.

Thank you for taking the time to read this information sheet.

This project was funded by the National Institute for Health Research PGfAR (project number RP-RG-1210-12017)

The REACH Study

Research Exploring physical Activity in Care Homes

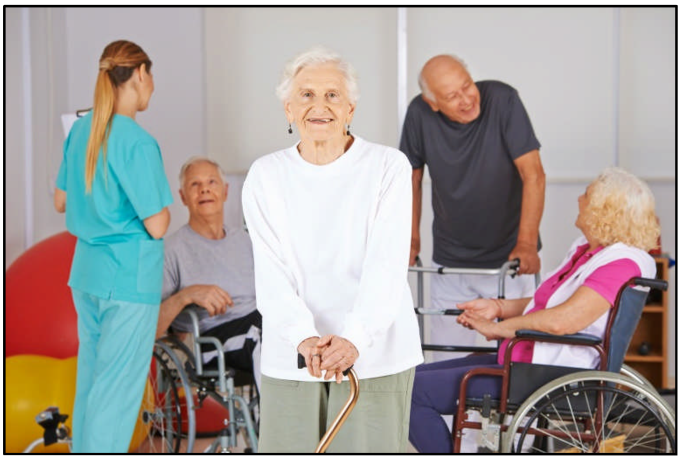

Resident Information Sheet

Invitation to take part in a research study

- We are inviting you to take part in a research project called the REACH study.
- Your care home has already agreed to take part.
- Before you decide whether to take part, we want you to understand what this involves.
- Please read this information leaflet carefully and take time to decide whether you would like to take part. Please ask if anything is unclear.
- You are free to decide whether or not to take part. If you choose not to it will not affect your care in any way.
- You can keep this information sheet to remind you about the study.

This project was funded by the National Institute for Health Research PGfAR (project number RP-RG-1210-12017)

How to contact us:

If you have any questions about this study, please contact:

Name of Researcher: <<insert>>
Telephone: <<insert>>

Or please contact the leads for this study:
<<insert>>

Or speak to any member of staff

This project was funded by the National Institute for Health Research PGfAR (project number RP-RG-1210-12017)

1. Why are we doing this study?

Research shows that increased movement can improve health and wellbeing. This study aims to look at how much activity people in care homes are able to do, and to see if there is a way to helpfully increase this.

Some care homes taking part in this study will use a new approach to increasing movement, whilst others will carry on as normal. . We will be able to compare information we collect from each home to help us to see if there are any differences between homes that use the new approach and those that don't.

The study is being carried out by Researchers at Bradford Teaching Hospitals NHS Trust and the University of Leeds. It also forms part of a PhD thesis looking at levels of movement in care homes, which is being undertaken by a Research Assistant working in the home.

2. What will happen in the study?

We are inviting 12 care homes in Yorkshire to take part. All homes will continue to care for residents as they would normally, but some will also be asked to use the new approach (intervention).

Study Researchers will visit each care home four times to find out how residents and members of staff are getting on. These visits will last a few weeks.

The Researchers will introduce themselves when they arrive and let you know what they are doing.

3. Why am I being asked to take part?

Your care home is involved in this study. We are inviting all residents at this care home to take part.

4. What will I be asked to do if I take part?

When the Researcher visits your care home s/he will ask if you would be willing to answer some questions. If you are happy to do this, it will

This project was funded by the National Institute for Health Research PGfAR (project number RP-RG-1210-12017)

take around 30 minutes on 3 or 4 occasions. S/he will also be collecting information from other residents and staff during his / her visit.

The Researcher will ask you some questions about your activity levels and about how you have been feeling.

S/he will also ask if you would be willing to wear an activity monitor. These are small (about the size of a matchbox), very light and are worn round the waist (like a belt). There is a picture of one below, and the Researcher will wear one herself to show you how it is done. The Researcher will ask you to wear this during waking hours, she will also help you put it on and take it off.

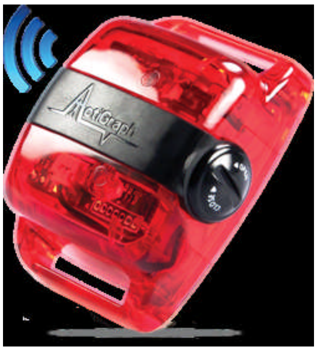

The monitors are completely harmless and just measure the distance you move - a bit like a mileage counter in a car. So, like in a car, they record the amount of movement you make over a period of time (e.g. when you are walking to the dining room, and then when you walk back to the sitting room).

5. What else will happen if I take part?

The Researcher will ask a member of staff who knows you well about how you have been getting on recently and will look at your records to get some information - for example, to see if you are taking any medicines or if you have seen the doctor recently.

The Researchers may also collect information from a central database (called the HSCIC) that shows when people have been to hospital.

If you are happy to take part please fill out and sign the consent form we have given you. The Researcher can help you with this and answer any questions you may have. You don't need to decide now and you should discuss it with family or friends if you wish.

This project was funded by the National Institute for Health Research PGfAR (project number RP-RG-1210-12017)

6. What will happen to the results of the study?

A summary of what we find out from this study will be sent to your care home and we will ask them to share this with all residents who took part in the study. The results would never include any information which would identify you or anyone else in the study in any way.

We will also share the results with as many people as possible through writing articles and reports and presenting at conferences and events.

7. Will my information be safe?

Yes. We will not record your name or any personal information on any of the information we collect or on any reports we write. We will keep any personal details about you, such as your name and date of birth, in a safe place. Only some members of the research team will be able to look at this information. Information you and care home staff tell us will be sent securely to the University of Leeds and the Bradford Institute of Health Research where it will be stored securely. It is possible that the information you provide for this study may be shared with other research teams to answer new research questions in the future. If this happened your information would be entirely anonymous.

8. What if I don’t want to take part?

You don’t have to take part if you don’t want to and you don’t have to give us a reason. It will not affect the care you receive.

If you are happy to take part now, but later decide to stop taking part, you can let us know by contacting us or asking your care home to do so. We will stop collecting information about you.

You can still take part in the study even if you later decide that you do not want to wear the activity monitor or speak to the Researcher. Just tell the Researcher you only want us to collect data from staff about

how you are getting on. Whatever you decide, this will not affect the care you receive.

9. What are the advantages and risks of taking part?
We hope that this study will help us to develop ways of increasing movement of residents in care homes in the future, but we cannot say that you will definitely see a difference yourself. The same would be true if you were not part of the study.

We do not expect that there will be any risks in taking part.

10. What if there is a problem?
A group of experts in people’s health and care has approved this research (number 15/EE/0125), and the care provided and research will be covered by normal insurance policies.

If you have any worries about this project you should speak to the Researcher, the lead for this research in this home, or any member of staff. If you remain concerned you can contact the PALS service (Patient Advise and Liaison Service, Bradford Royal Infirmary, Tel: 01274 364021)

If you are harmed by taking part in this research project, there are no special compensation arrangements. If you decide to take legal action you may have to pay for it. Any claims will be subject to UK law and must be brought in the UK.

If the Researchers see any practice they feel is abusive or neglectful then this will be reported and investigated to see if any further action needs to be taken.

11. Questions
If you have any questions or would like more information, please speak to a member of staff, who will contact the Researcher.

If you would like further information about research in general, the UK Clinical Research Collaboration (a partnership of organisations

This project was funded by the National Institute for Health Research PGfAR (project number RP-RG-1210-12017)

working together on research in the UK) have published a booklet entitled 'Understanding Clinical Trials'. Contact UKCRC: Tel: 0207 670 5452; website www.ukcrc.org.

In Summary

You will be asked to complete a consent form to confirm you will take part in the study.

The Researcher will sit and chat to you about the physical activity monitors.

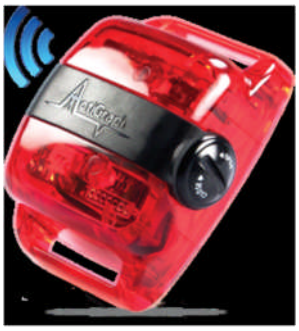

S/he will also see how you are getting on generally.

S/he will fill in some forms; you will not have to do any writing.

The Researcher will chat to a staff member who knows you well to ask how you are getting on.

The Researchers will visit you three more times to see how you are doing and complete some more forms. S/he will ask you to wear the activity monitor if you're happy to do so.

(4 Researcher Visits over 9 months)

Thank you for taking the time to read this information sheet.

This project was funded by the National Institute for Health Research PGfAR (project number RP-RG-1210-12017)

UNIVERSITY OF LEEDS

REACH RESEARCH IN ENHANCING PHYSICAL ACTIVITY IN CARE HOMES

ctru

Bradford Teaching Hospitals NHS Foundation Trust

BRADFORD INSTITUTE FOR HEALTH RESEARCH

To be completed by REACH Researcher

Resident Trial No: _ _ _ _ _ _   Initials: _ _ _ _   DOB: _ _/_ _/_ _ _ _

Name of Care Home:…………………………………………………………………….

The REACH Study

Research **E**xploring physical **A**ctivity in **C**are **H**omes

Resident Consent Form

Please read the statements below and sign at the bottom if you agree to take part.

1.	I confirm that I have read the information sheet dated………………….. (Version ……….) for the REACH study and have had the chance to ask questions.
2.	I understand that taking part in this study is my choice and that I am free to withdraw at any time without my care being affected.
3.	I understand that even if I withdraw from the study, the data collected from me up to that point will be used in analysing the results of the study.
4.	I understand that relevant sections of my medical/care records and data collected during the course of the study may be looked at by members of the research team, responsible individuals from the NHS or regulatory authorities. I give permission for these individuals to have access to my records.
5.	I understand that the information held and maintained by The Health and Social Care Information Centre (HSCIC) and other NHS bodies may be used to provide information about my health status. E.g. If I have been to hospital.
6.	I agree to allow any information or results arising from this study to be used for further research upon the understanding that my identity will remain anonymous.
7.	I agree for my details and a copy of this consent form (which will include my name and date of birth) to be stored by the Research Office (at the University of Leeds) for the purposes of this study.
8.	I agree to take part in the study.

This project was funded by the National Institute for Health Research PGfAR (project number RP-RG-1210-12017)

To be completed by REACH Researcher

Resident Trial No: _ _ _ _ _ _ Initials: _ _ _ _ DOB: _ _/_ _/_ _ _ _

Name of Care Home:……………………………………………………………………

Participant

Signature ………………………………………………………………

Name (block capitals) …………………………………….

Date ………………………….

Witness (if needed)

Signature ……………………………………………………………

Name (block capitals) ………………………………………………

Date …………………………….

Researcher

I have explained the study to the above named participant and he/she has indicated his/her willingness to participate.

Signature ………………………………………………………………

Name (block capitals) ………………………………………………

Date ………………………….

(Original to be sent to the Research Office; 1 copy for participant, 1 copy for care home records)

This project was funded by the National Institute for Health Research PGfAR (project number RP-RG-1210-12017)

The REACH Study

Research Exploring physical Activity in Care Homes

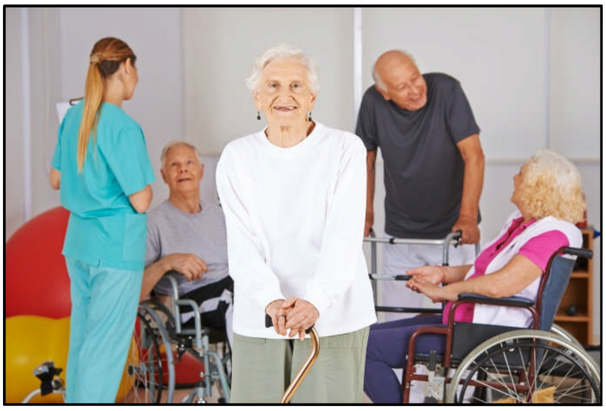

Personal Consultee Information Sheet

Invitation to be a personal consultee

- We are inviting you to take the role of personal consultee for your relative / friend who has been invited to take part in a research project called the REACH study.
- His/her care home has already agreed to take part.
- This leaflet explains what a personal consultee is.
- Before you decide whether or not you are willing to be a personal consultee for your relative / friend, we want to explain what this involves.
- Please read this information leaflet carefully and take time to decide whether you are willing and able to take on this role.
- You are free to decide whether or not to take on this role. If you choose not to it will not affect your relative / friend's care in any way.
- If you decide to be a personal consultee you should keep this information sheet for future reference.
- Ask us if anything is unclear, or if you would like more information.

How to contact us:

If you have any questions about this study, please contact:

Name of Researcher: <<insert>>
Telephone: <<insert>>
Or please contact the leads for this study:

<<insert>>

This project was funded by the National Institute for Health Research PGfAR (project number RP-RG-1210-12017)

1. What is a personal consultee?

A personal consultee is a relative or close friend of a resident living in a care home. A personal consultee gives the researchers advice about whether the resident should participate in a study when the resident is not able to make that decision for themselves. A resident is sometimes unable to make this decision for him or herself if he/she has memory problems.

2. What does a personal consultee need to do?

A personal consultee provides **advice** to the researcher about their relative/friend's wishes and feelings in relation to the project and whether he or she should be part of the study.

A personal consultee provides advice based on their knowledge of their relative/friend and gives their opinion on what their relative/friend's past and present wishes and feelings would have been about taking part in the study. A personal consultee is **not** asked to provide consent for or on behalf of their relative/friend.

At any stage during the study, a personal consultee should also advise the researchers if they feel their relative/friend no longer wishes to take part in the research.

3. Why have I been asked to be a personal consultee?

Your relative/friend's care home is involved in this study. The care home manager and a researcher have spoken with your relative/friend, because we would like to invite them to take part.

After talking to your relative/friend we think they may not have the capacity to make this decision for themselves. However, they did not indicate that they did not want to take part. We would therefore like to appoint a 'personal consultee' who knows the resident well and will talk to them and speak on their behalf about their wish to take part in the study.

4. What if I don't feel able to take on this role?

If you feel unable to give advice about this, please say so. This will not affect your relative/friend's care, or whether or not they take part in the study.

If we have not heard back from you within 10 days, a member of the care home team will send a reminder. If we've not heard back after a further 10 days, or if you tell us that you are not able to take on this role we will ask your relative/friend and the home's management team to help us identify another relative/friend of the resident, who could take on this role. You can also suggest someone we might approach.

This project was funded by the National Institute for Health Research PGfAR (project number RP-RG-1210-12017)

If there is no-one else able or willing to take on this role then we will ask a staff member who knows your relative/friend well, but who is not involved in the research, to help us find out about your relative/friend's wishes and advise us on these. They may contact you and other relatives/friends to help them.

5. If I agree to take on this role, what do I need to do?

We would like you to read the study information sheet provided, which tells you what being part of the study would mean for your relative/friend. Using what you know of the wishes and feelings of your relative/friend, please advise us on whether you feel he/she would have agreed to join the study, if he/she had been able to decide for him/herself.

Please base your advice on your knowledge of your relative/friend and their past and present views or feelings, not on your own views of research in general or this project. You should try to seek the views of your relative/friend, if appropriate, and also the views of other family or friends in helping give this advice.

After these discussions we would like you to complete the attached assent form indicating whether you feel your relative/friend would or would not have wished to participate.

If your relative / friend does take part in the study and during the course of the study you feel they have changed their mind about taking part, or that taking part is causing them distress, then you should tell one of the researchers or a member of care home staff who will let the researchers know.

We will ask for some contact details from you in case we need to contact you in the future regarding your relative / friend's ongoing participation in the study.

6. What if I feel my friend/relative does not want to take part?

There is no obligation to take part. If after consulting with your relative/friend you feel they do not wish to take part, please let the researcher know so that they are aware not to approach another relative or friend for their help. You may provide a reason for declining participation if you wish although this is optional. If they agree now but then change their mind at a later date you can contact us and we will make sure we don't involve them in any more research processes (as described in the study information sheet). Whatever you advise us, this will not affect the care they receive.

7. Questions

If you have any questions or would like more information, you can speak to the Researcher, whose details can be found on page 1 of this information sheet.
If you would like further information about research in general, the UK Clinical Research Collaboration (a partnership of organisations working together on research

in the UK) have published a booklet entitled ‘Understanding Clinical Trials’. Contact UKCRC: Tel: 0207 670 5452; website www.ukcrc.org.

Thank you for taking the time to read this information sheet.

This project was funded by the National Institute for Health Research PGfAR (project number RP-RG-1210-12017)

UNIVERSITY OF LEEDS

REACH RESEARCH IN ENHANCING PHYSICAL ACTIVITY IN CARE HOMES

ctru

Bradford Teaching Hospitals NHS Foundation Trust

BRADFORD INSTITUTE FOR HEALTH RESEARCH

To be completed by REACH Researcher
Resident Trial No: _ _ _ _ _ _   Initials: _ _ _ _   DOB: _ _/_ _/_ _ _ _
Name of Care Home:…………………………………………………………………….

The REACH Study

Research Exploring physical Activity in Care Homes

Personal Consultee Declaration Form

Completed in relation to the wishes of:

Resident name (my relative/friend):……………………………

Care home:…………………………………………………………

OPTION 1 – Agreeing to participation
If you agree that your relative/friend would want to take part in this study, please read each statement and sign and date at the bottom of the form.

1.	I confirm that I have read the information sheet dated………………….. (Version ……….) for the REACH study and have had the chance to ask questions.
2.	I understand that my relative/friend's (the resident) participation in this study is voluntary and that he/she is free to withdraw at any time without their care being affected.
3.	I understand that even if my relative/friend (the resident) withdraws from the study, the data collected from him/her up to that point will be used in analysing the results of the study.
4.	I understand that relevant sections of my relative/friend's medical/care records and data collected during the course of the study may be looked at by members of the research team, responsible individuals from the NHS or regulatory authorities.
5.	I understand that the information held and maintained by The Health and Social Care Information Centre (HSCIC) and other NHS bodies may be used to provide information about my relative/friend's health status. E.g. If they have been to hospital.
6.	I agree to allow any information or results arising from this study to be used for further research upon the understanding that it will remain anonymous.
7.	I agree for my relative/friend's details and a copy of this declaration form to be stored by the Research Office (at the University of Leeds) for the purposes of this study.
8.	I agree for my name and contact information to be held at the Research Office (at the University of Leeds) for the purposes of this study.
9.	I agree to my relative/friend (the resident) taking part in the study.

This project was funded by the National Institute for Health Research PGfAR (project number RP-RG-1210-12017)

OPTION 2 – Not agreeing to participation

If you feel your relative/friend **would not** have wanted to take part in this study, please tick this box ☐

If you would like to provide a reason for declining study participation please complete the question below – **Please note this is optional:**

I do not feel my relative/friend would like to take part in this study because:

☐ I do not feel my relative/friend is well enough to take part

☐ I do not think my relative or friend would choose to take part in the study if they were able to decide for themselves

☐ Other reason (please specify)……………………………………………………………………………………
……………………………………………………………………………………………
……………………………………………………………………………………………

Please sign and date at the bottom of the form and return it to the researcher

OPTION 3 - Uncertain

If you feel you are **unable to make a judgment** on your relative/friend's interest in taking part in this study please tick this box ☐ , sign and date at the bottom of the form and return to the researcher.

Note: if we do not hear back from you, then we will contact a member of staff within the care home to make an assessment of your relative/friend's views on participation in this study.

Please sign and return this form in the pre-paid envelope provided.

Thank you.
Personal consultee

Signature ……………………………………………………………

Name (block capitals) ……………………………………………….

Date ………………………….

Relationship to resident ……………………………………………..

Address……………………………………………………………………………………………

……………………………………………………………………………………………………

Telephone Number…………………………………………………..

This project was funded by the National Institute for Health Research PGfAR (project number RP-RG-1210-12017)

Researcher

I have explained the study to the above named consultee and he/she has indicated that in his/her opinion their relative/friend would/would not (delete as appropriate) be willing to participate.

Signature ………………………………………………………………

Name (block capitals) ………………………………………………

Date ………………………….

(Original to be sent to the Research Office; 1 copy for consultee, 1 copy for care home records)

This project was funded by the National Institute for Health Research PGfAR (project number RP-RG-1210-12017)

The REACH Study

Research **E**xploring physical **A**ctivity in **C**are **H**omes

Staff Nominated Consultee Information Sheet

Invitation to be a nominated consultee

- We are inviting you to take the role of *nominated consultee* for a resident from your care home who has been invited to take part in the REACH study.
- Before you decide whether or not you are willing to take on this role, we want to explain what this involves.
- Please read this information leaflet carefully and take time to decide whether you are willing to take on this role.
- You are free to decide whether or not to take this role. If you choose not, that's not a problem at all.
- If you decide to take on this role you should keep this information sheet for future reference.
- Ask us if anything is unclear, or if you would like more information.

How to contact us:

If you have any questions about this study, please contact:

Name of Researcher: <<insert>>
Telephone: <<insert>>

Or please contact the leads for this study:

<<insert>>

This project was funded by the National Institute for Health Research PGfAR (project number RP-RG-1210-12017)

1. What is a nominated consultee?

A nominated consultee is a staff member/other professional who advises on a resident's possible participation in a research project, when the person has been assessed to lack capacity to make an informed decision themselves.

They are appointed where the potential participant does not have a family member or close friend who is able or willing to act as a consultee.

2. What does a nominated consultee need to do?

A nominated consultee provides **advice** to the researcher about a resident's wishes and feelings in relation to the project and whether or not he or she should be part of the study.

A nominated consultee provides advice on whether, in their opinion, based on their knowledge of the resident, the resident would be content to take part, or whether participating might upset them.

You would also be asked to consider what the resident's past and present wishes and feelings would be about taking part in the study. A nominated consultee is **not** asked to provide consent for or on behalf of the potential participant.

At any stage during the study, a nominated consultee should also advise the researchers if they feel the participant no longer wishes to take part in the research.

A nominated consultee is not allowed to be involved in any way in the research taking place in the care setting as this could influence their decision about resident involvement.

3. Who are you asking me to be a nominated consultee for?

We believe one or more residents within the care home in which you work are unable to decide for themselves about taking part in this study. We are therefore seeking a nominated consultee to provide advice on whether you feel the resident(s) would have wanted to take part. The name of the resident you are being asked to advise on is at the top of the enclosed declaration form(s).

4. What if I don't feel able to take on this role?

If you feel unable to give advice about this, please say so. This will not affect your employment or the resident's future involvement in the study. We will work with the home's senior management team to identify another staff member to approach with regards to this role.

This project was funded by the National Institute for Health Research PGfAR (project number RP-RG-1210-12017)

5. If I agree to take on this role, what do I need to do?
We would like you to read the study information sheet provided, which tells you what being part of the study would mean for the resident. We would then like you to consider what you know of the wishes and feelings of the resident to help you decide how they might feel about taking part in the study.

We would like to know whether or not you feel he/she would have agreed to join the study, if he/she had been able to decide. Your advice should be based on your knowledge of the resident and their past and present views or feelings, not on your own views of research in general or this project.

In deciding what you think the resident's wishes would be if they had capacity, you should attempt to seek the views of the person, and also the views of their family or friends who may be unwilling or unable to act as a consultee.

You may, where appropriate, also seek the views of other colleagues who have an interest in the person's welfare, **but who won't be involved in the research themselves**.

After appropriate consultation we would like you to complete the attached declaration form indicating whether you feel the named person would or would not have wished to participate.

If the resident does take part in the study and during the course of the study you feel they have changed their mind about taking part, or that it is causing them distress then you should tell one of the researchers.

6. Questions
If you have any questions or would like more information, you can speak to the Researcher, whose details can be found on page 1 of this information sheet.
If you would like further information about research in general, the UK Clinical Research Collaboration (a partnership of organisations working together on research in the UK) have published a booklet entitled 'Understanding Clinical Trials'. Contact UKCRC: Tel: 0207 670 5452; website www.ukcrc.org.

Thank you for taking the time to read this information sheet.

This project was funded by the National Institute for Health Research PGfAR (project number RP-RG-1210-12017)

To be completed by REACH Researcher

Resident Trial No: _ _ _ _ _ _ Initials: _ _ _ _ DOB: _ _/_ _/_ _ _ _

Name of Care Home:…………………………………………………………………

The REACH Study

Research **E**xploring physical **A**ctivity in **C**are **H**omes

Nominated Consultee Declaration Form

Completed in relation to the wishes of:

Resident name:…………………………………………………

Care home:…………………………………………………….

OPTION 1 – Agreeing to participation

If you agree that the resident would want to take part in this study, please read each statement and then sign and date at the bottom of the form.

1.	I confirm that I have read the information sheet dated………………….. (Version ……….) for the REACH study and have had the chance to ask questions.
2.	I understand that the named resident's participation in this study is voluntary and that he/she is free to withdraw at any time without their care being affected.
3.	I understand that even if the named resident withdraws from the study, the data collected from him/her up to that point will be used in analysing the results of the study.
4.	I understand that relevant sections of the named resident's medical/care records and data collected during the course of the study may be looked at by members of the research team, responsible individuals from the NHS or regulatory authorities.
5.	I understand that the information held and maintained by The Health and Social Care Information Centre and other NHS bodies may be used to provide information about the named resident's health status. E.g. Hospital admissions.
6.	I agree to allow any information or results arising from this study to be used for further research upon the understanding that it will remain anonymous.
7.	I agree for the named resident's details and a copy of this declaration form to be stored by the Research Office (at the University of Leeds) for the purposes of this study.
8.	I agree to the named resident taking part in the study.

This project was funded by the National Institute for Health Research PGfAR (project number RP-RG-1210-12017)

To be completed by REACH Researcher

Resident Trial No: _ _ _ _ _ _ Initials: _ _ _ _ DOB: _ _/_ _/_ _ _ _

Name of Care Home:……………………………………………………………………

OPTION 2 – Not agreeing to participation

If you feel the resident **would not** have wanted to take part in this study, please tick this box ☐ , sign and date at the bottom of the form and return it to the researcher.

OPTION 3 - Uncertain

If you feel you are **unable to make a judgment** on the resident's interest in taking part in this study please tick this box ☐ , sign and date at the bottom of the form and return to the researcher.

Nominated consultee

Signature ……………………………………………………………

Name (block capitals) ………………………………………

Date ………………………….

Relationship to resident…………………………………….

Researcher

I have explained the study to the above named consultee and he/she has indicated that in his/her opinion their relative/friend would/would not (delete as appropriate) be willing to participate.

Signature ……………………………………………………………

Name (block capitals) …………………………………………………

Date ………………………….

(Original to be sent to the Research Office; 1 copy for consultee, 1 copy for care home records)

This project was funded by the National Institute for Health Research PGfAR (project number RP-RG-1210-12017)

The REACH Study

Research **E**xploring physical **A**ctivity in **C**are **H**omes

Staff Information Sheet

Invitation to participate

- We are inviting you to take part in a research project called the REACH study.
- Before you decide whether or not you wish to participate we would like to explain why the research is being done and what it will involve.
- Please read this information leaflet carefully and take time to decide whether or not to take part.
- You are free to decide whether or not to take part. If you choose not to, this will not affect your employment in any way.
- If you decide to take part, you should keep this information sheet for future reference
- Ask us if anything is unclear, or if you would like more information.

(Please turn over)

Contents

1. Why are we doing this study?
2. Who is carrying out the study?
3. What will happen in the study and what will I need to do?
4. Who will see the information I provide?
5. What if I don't want to take part?
6. What are the advantages and risks of taking part?
7. More information about taking part
8. Questions

How to contact us:

If you have any questions about this study, please contact:
Name of Researcher: <<insert>>
Telephone: <<insert>>

Or please contact the leads for this study:

<<insert>>

This project was funded by the National Institute for Health Research PGfAR (project number RP-RG-1210-12017)

1. Why are we doing this study?

Research shows that increased movement can improve health and wellbeing. This study aims to look at how much activity people in care homes are able to undertake, and to see if there is a way to helpfully increase this.

Some care homes taking part in this study will use a new approach to increasing movement, whilst others will carry on as normal. We will be able to compare information we collect from each home to help us to see if there are any differences between homes that use the new approach and those that don't.

2. Who is carrying out the study?

The study is being carried out by researchers at Bradford Teaching Hospitals NHS Trust and the University of Leeds. It also forms part of a PhD thesis looking at measuring levels of movement in care homes, which is being undertaken by a Research Assistant who will be working with the care homes.

3. What will happen in the study and what will I need to do?

*(The **black** writing describes what will happen and the **blue** writing highlights the parts we would like you and / or other members of staff to help with.)*

Involving care homes in Yorkshire
We plan to ask 12 care homes across Yorkshire to take part in this research. Six of the care homes will carry on delivering care as usual, while six will be randomly chosen to use the new approach.

We will collect information from all 12 care homes so we can find out how residents and staff found the new approach – and so we can compare those homes that use the intervention with those that don't.

Resident consent
Before it is decided whether or not a care home will use the new approach, residents will be asked to provide their consent to be involved in this study. We may also ask a relative or staff member to act as a 'consultee' to provide agreement on a resident's behalf if they are unable to make the decision themselves. If they give consent (or assent) to be involved, this will mean a researcher from the study will visit to collect some information about their physical activity and ask them some questions on four occasions over 9 months. We will not involve anyone in the study who has not consented to this and they can change their mind about taking part at any time.

This project was funded by the National Institute for Health Research PGfAR (project number RP-RG-1210-12017)

Involving staff as ‘consultees’

Where a resident isn’t able to make a decision for themselves about taking part in the study (e.g. if they have problems with memory or understanding), we will try to contact a relative or friend who could help them make that decision. (A relative or friend would be known as a ‘personal consultee’). Where a relative or friend isn’t available to help in this way, we may ask you to be a consultee for a resident. By doing this you would be thinking about whether you feel they would want to take part if they *were* able to make the decision themselves. If you are asked to do this, we would give you a separate information sheet which explains in more detail what it means to be a consultee. You wouldn’t have to do this if you didn’t want to.

Care Home ‘Allocation’ - Usual Care or ‘MoveMore’ (the intervention)

Once all residents have had the opportunity to join the study, care homes will be allocated to use the ‘MoveMore’ intervention or continue with ‘usual care’ (i.e. carrying on doing what you would normally do). The best way to compare the two approaches is to decide by chance (‘randomly’) who gets which one. So in this study your care home will be either part of the ‘usual care’ group of homes or the ’MoveMore’ group of homes. At the end of the study these two groups can be compared to see whether one approach is more helpful than the other.
The information we collect from both the ’MoveMore’ and ‘usual care’ homes is equally important for the research. We would compensate you for any time you spend providing information to the researcher.

‘MoveMore’ (the intervention)

’MoveMore’ is a package of information, suggestions and practical ideas which can be used to help care homes to introduce more movement into residents’ every day care home life. The package is based on a review of what has been tried previously in other studies, through observations in other care homes, and by discussing how this might work best with other care home residents and staff.

If your care home is allocated to use ‘MoveMore’, members of the research team will arrange three workshop sessions with a group of staff members to explain the package and how to tailor this to fit in with your home’s every day activities. These workshop sessions will be audio recorded; however, staff members have the right to decline this if they wish. After the workshop sessions it would be up to staff members to find the best ways of making changes to increase movement amongst residents. It will be about making small changes to every day routines to help residents to be more active. So it will be about doing things a bit differently, rather than doing more work.

We will ask you to make a note of what changes are made so we can see what works well and what is less helpful.

This project was funded by the National Institute for Health Research PGfAR (project number RP-RG-1210-12017)

Researcher visits
All research needs information (data) to be able to report results at the end of the study. For this study a researcher will visit your care home to collect information from residents about how they are doing, as well as to collect some information from staff members and from the care home records.

The study researcher will visit each care home to collect information four times over 9 months (at the start, 3 months later, 6 months later and 9 months later). One researcher will visit the care home for around two to four weeks to collect this information at each time point.

As a number of the residents living in this home will be taking part in this study we need to gather information about how they are doing at each researcher visit.

We will invite residents to assist us in the study by wearing activity monitors (a small device very similar to a pedometer) at each of the four researcher visits. This will enable us to record levels of movement among residents. The activity monitors will be worn round the waist during the day for around 2 to 5 days at each of the four researcher visits. We will also offer training to all staff members in how to use the activity monitors as we may ask you to assist in putting them on/taking them off; we may also ask for a record to be kept of how long each resident has worn their monitor.

We will ask residents to fill in some questionnaires with the researcher at each visit. These ask about their quality of life and how they have been feeling. We will talk to the resident where possible and ask them how they are doing. However, because of communication and memory difficulties not all residents will be able to give us information and we may therefore ask staff members who know the resident(s) well to help with this.

We will also ask some staff to help by providing information about residents' physical activity and mobility.

If you are happy to provide data relating to a resident(s) you know well, the researcher will sit with you and ask you questions about the resident or ask you to complete a questionnaire on behalf of the resident. This should take no more than 30 minutes for each resident. We can do this at a time convenient to you during the time the researcher visits. The home's management team has agreed staff can fill out these forms with a researcher during your normal working duties.

We will ask all staff members (if you are willing) to complete a questionnaire about themselves which will take about 5-10 minutes.

This project was funded by the National Institute for Health Research PGfAR (project number RP-RG-1210-12017)

The researcher may have some discussion sessions (focus groups) with a small group of staff and residents to explore what aspects of the study worked well and which aspects could be improved.

4. Who will see the information I provide?

Only the research team will see any information collected about you or the resident and it will not be shared with anyone you work with, the resident or their family.

Completion of all questionnaires is confidential, which means we won't record your name alongside the answers you give and care home managers/colleagues will not see it.

If you are asked to provide any data relating to a resident(s) you know well, and you are happy to do this, we will ask for some basic information (e.g. name and job role) so we can contact you again at future data collection points if required.

5. What if I don't want to take part?

You don't have to take part if you don't want to. You are free to decide whether or not you wish to provide data about yourself or a resident you know well. If you decide not to take part you do not have to give a reason and this will not affect your employment in any way.

Staff questionnaires will be circulated to all staff members at each time point, however, if you do not wish to provide this data, do not return the questionnaire.

6. What are the advantages and disadvantages of taking part?

Taking part will help us gain an understanding of how the new approach can be used to increase physical activity in care homes and so may help residents in the future.

We do not expect there will be any risks in taking part.

7. More information about taking part

What will happen if I do not want to carry on providing data?

You can choose not to provide data about yourself at any time point by simply not returning the staff questionnaire. You can also decide against providing data relating to a resident at any time point, this will not affect your employment in any way. In these instances the researcher will ask the management team to identify another staff member who knows the resident well. Any information already collected from you for the study will remain on file and will be included in the final study analysis. At the end of the study data we collect from you and from residents will be securely

This project was funded by the National Institute for Health Research PGfAR (project number RP-RG-1210-12017)

stored for a minimum of 5 years. Arrangements for confidential destruction will then be made.

Will my taking part be kept confidential?

If you decide to provide data for the REACH study, the information collected about you, your home, your staff and the residents during the course of the study will be kept strictly confidential. The information collected will be recorded on paper forms and sent securely from your care home to the University of Leeds and the Bradford Institute of Health Research. This information will be securely stored at the Research Office at Leeds University on paper and electronically, under the provisions of the 1998 Data Protection Act. At the end of the study, your data will be securely archived for a minimum of 5 years. Arrangements for confidential destruction will then be made.

It is possible that the information you provide for this study may be shared with other research teams to answer new research questions in the future. If this happens the information would be anonymised, meaning your care home name, staff and resident names would never be included, so that no-one would be able to identify you or any study participants from it. Other research teams would not be given any personal details about who participated in the study.

What will happen to the results of the study?

The results of the study will be shared with people living in care homes and their families, care provider organisations and their staff, policy makers, researchers and the general public. We will share the results through writing articles for magazines and journals, through speaking at conferences and other public events, through producing information leaflets and through the web-pages of the organisations involved. No information that might identify you will ever be included in information we share about the study results.

We will produce a specific information leaflet about the results of the study for staff. These will be sent to the care home. If you leave the care home before the study ends then you can ask for us to send you a copy to an address of your choice when the study ends.

What if there is a problem?

If you have a concern about any aspect of this study, you should ask to speak with the Researcher who will do their best to answer your questions. If you remain unhappy you may wish to contact the PALS service (Patient Advise and Liaison Service at Bradford Royal Infirmary, Tel: 01274 364021).

This project was funded by the National Institute for Health Research PGfAR (project number RP-RG-1210-12017)

If you are harmed by taking part in this research project, there are no special compensation arrangements. If you decide to take legal action you may have to pay for it. Any claims will be subject to UK law and must be brought in the UK.

If the Researchers see any practice they feel is abusive or neglectful then this will be reported and investigated to see if any further action needs to be taken.

Who is organising, funding and reviewing the research?
The study is being organised and supervised by Bradford Teaching Hospitals and the University of Leeds. It is funded by research funding from the Department of Health.
All research is looked at by an independent group of people called a Research Ethics Committee to protect the safety, rights, wellbeing and dignity of those taking part. This study has been reviewed and approved by the East of England - Norfolk Research Ethics Committee (REC Reference: 15/EE/0125).

8. Questions?

If you have any questions or would like more information, you can speak to the Researcher, whose details can be found on page 1 of this information sheet.
If you would like further information about research in general, the UK Clinical Research Collaboration (a partnership of organisations working together on research in the UK) have published a booklet entitled 'Understanding Clinical Trials'. Contact UKCRC: Tel: 0207 670 5452; website www.ukcrc.org.

Thank you for taking the time to read this information sheet.

This project was funded by the National Institute for Health Research PGfAR (project number RP-RG-1210-12017)

UNIVERSITY OF LEEDS

REACH RESEARCH IN ENHANCING PHYSICAL ACTIVITY IN CARE HOMES

ctru

Bradford Teaching Hospitals NHS Foundation Trust

BRADFORD INSTITUTE FOR HEALTH RESEARCH

The REACH Study

Research **E**xploring physical **A**ctivity in **C**are **H**omes

Resident Information Sheet - Interviews

<Insert picture here>

- We are inviting you to take part in an interview or discussion group as part of the REACH study.
- You and your care home are already a part of the REACH study.
- Before you decide whether to take part, we want you to understand what this involves.
- Please read this information leaflet carefully and take time to decide whether you would like to take part.
- Please ask if anything is unclear.
- You are free to decide whether or not to take part. If you choose not to it will not affect your care in any way.
- You can keep this information sheet as a reminder.

How to contact us:

If you have any questions about this study, please contact:

Name of Researcher: <<insert>>
Telephone: <<insert>>

Or please contact the leads for this study:
<<insert>>

This project was funded by the National Institute for Health Research PGfAR (project number RP-RG-1210-12017)

1. Why are we doing research interviews?
The aim of the interviews is to get some feedback from both residents and staff members on which parts of the REACH study are working well and those that may not be working so well.

We are asking some residents and some staff members if they might be willing to discuss their thoughts about the study with the researcher.

2. What will happen?
If you agree to take part, the researcher will arrange a convenient time with you to have a chat about your experiences in the REACH study.

This will take about one hour and will take place at a time and in a private location of your choice. If you wish to pause or stop the discussion at any point, you will be able to do so.

The discussion you have with the researcher will be recorded using a Dictaphone/Tape recorder.

The researcher will ask you some questions about how you are finding being part of the REACH study. You will have the opportunity to discuss any aspects that you like and those that you are unsure about.

3. Will the information I give be safe?
Yes. All answers will be anonymous and will only be shared with the research team. Other staff members and other residents will not have access to the answers you give. Your name will be held separately from the interview recording and any transcriptions (typing up the interview onto a computer) that are made.

4. What will happen to the recording of the interview?
The Dictaphone or tape recorded interview will be transported securely to Bradford Institute of Health Research (where the researcher is based). It will be stored safely in the research offices in Bradford, and only members of the research team will be able to access it.

This project was funded by the National Institute for Health Research PGfAR (project number RP-RG-1210-12017)

All the interviews that we record as part of the study (from other residents and staff across the participating care homes) will be analysed by the research team. Any results from the interviews will be entirely anonymous - which means they would not identify you in any way.

The results of the study as a whole (all the study information we collect, as well as the interviews) will be published. A summary will be sent to your care home and we will ask them to share these with all residents and staff who took part in the study.

5. What if I don't want to take part?
You don't have to take part in the research interview if you don't want to and you don't have to give us a reason. It will not affect your care in any way.

6. What are the advantages and risks of taking part?
Your feedback may help us to understand how we can improve the way we run the study if we continue working with other residents and care homes. We do not anticipate there will be any risk in taking part in these research interviews.

7. What if there is a problem?
If you have any worries about this project you should speak to the researcher, the lead for this research in your care home or a member of staff. If you remain concerned you can contact the PALS service (Patient Advice and Liaison Service, Bradford Royal Infirmary, Tel: 01274 364021).

8. Questions
If you have any questions or would like more information, please ask the Researcher directly.

If you would like further information about research in general, the UK Clinical Research Collaboration (a partnership of organisations working together on research in the UK) have published a booklet entitled 'Understanding Clinical Trials'. Contact UKCRC: Tel: 0207 670 5452; website www.ukcrc.org.

This project was funded by the National Institute for Health Research PGfAR (project number RP-RG-1210-12017)

Thank you for taking the time to read this information sheet.

To be completed by REACH Researcher<br>Resident Trial No: _ _ _ _ _ _ Initials: _ _ _ _ DOB: _ _/_ _/_ _ _ _<br>Name of Care Home:……………………………………………………………………

The REACH Study

Research **E**xploring physical **A**ctivity in **C**are **H**omes

Resident Consent Form - Interviews

Please read the statements below and sign at the bottom if you agree to take part.

1.	I confirm that I have read the information sheet dated………………….. (Version ……….) and have had the chance to ask questions.
2.	I understand that taking part in the research interviews is my choice and that I am free to withdraw at any time without my care being affected.
3.	I understand that even if I withdraw from the study, the data collected from me up to that point will be used in analysing the results of the study.
4.	I understand that the interview sessions will be recorded, transferred and stored securely at the Bradford Institute of Health Research.
5.	I agree for my details and a copy of this consent form (which will include my name and date of birth) to be stored by the Research Office (at the University of Leeds) for the purposes of this study.
6.	I agree to take part in the study interviews.

This project was funded by the National Institute for Health Research PGfAR (project number RP-RG-1210-12017)

Participant

Signature …………………………………………………………………

Name (block capitals) ……………………………………….

Date ………………………….

Witness (if needed)

Signature ……………………………………………………………….

Name (block capitals) ………………………………………………….

Date …………………………….

Researcher

I have explained the research interviews to the above named resident and he/she has indicated his/her willingness to participate.

Signature ………………………………………………………………….

Name (block capitals) ………………………………………………….

Date ………………………….

(Original to be sent to BIHR; 1 copy for participant, 1 copy for care home records)

This project was funded by the National Institute for Health Research PGfAR (project number RP-RG-1210-12017)

The REACH Study

Research Exploring physical Activity in Care Homes

<Insert picture here>

Staff Information Sheet - Interviews

- We are inviting you to take part in an interview or discussion group as part of the REACH study.
- Your care home is already a part of the REACH study.
- Before you decide whether to take part, we want you to understand what this involves.
- Please read this information leaflet carefully and take time to decide whether you would like to take part.
- Please ask if anything is unclear.
- You are free to decide whether or not to take part. If you choose not to it will not affect your employment in any way.
- You can keep this information sheet as a reminder.

How to contact us:

If you have any questions about this study, please contact:

Name of Researcher: <<insert>>
Telephone: <<insert>>

Or please contact the leads for this study:
<<insert>>

This project was funded by the National Institute for Health Research PGfAR (project number RP-RG-1210-12017)

1. Why are we doing research interviews?
The aim of the interviews is to gain feedback from both residents and staff members on which parts of the REACH study are working well and those that may not be working so well.

We are asking some staff members and some residents if they might be willing to discuss their thoughts about the study with the researcher.

2. What will happen?
If you agree to take part, the researcher will arrange a convenient time with you to have a discussion about your experiences in the REACH study.

This will take about one hour and will take place at a time and in a private location of your choice. If you wish to pause or stop the discussion at any point, you will be able to do so.

The discussion with the researcher will be recorded using a Dictaphone/Tape recorder.

The researcher will ask you some questions about how you are finding being part of the REACH study. You will have the opportunity to discuss any aspects that you like and those that you are unsure about.

3. Will the information I give be safe?
Yes. All answers will be anonymous and will only be shared with the research team. Other staff members and other residents will not have access to the answers you give. Your name will be held separately from the interview recording and any transcriptions (typing up the interview onto a computer) that are made.

4. What will happen to the recording of the interview?
The Dictaphone or tape recorded interview will be transported securely to Bradford Institute of Health Research (where the researcher is based). It will be stored safely in the research offices in Bradford, and only members of the research team will be able to access it.

This project was funded by the National Institute for Health Research PGfAR (project number RP-RG-1210-12017)

All the interviews that we record as part of the study (from other staff and residents across the participating care homes) will be analysed by the research team. Any results from the interviews will be entirely anonymous – which means they would not identify you in any way.

The results of the study as a whole (all the study information we collect, as well as the interviews) will be published. A summary will be sent to your care home and we will ask them to share these with all residents and staff who took part in the study.

5. What if I don't want to take part?
You don't have to take part in the research interview if you don't want to and you don't have to give us a reason. It will not affect your employment in any way.

6. What are the advantages and risks of taking part?
Your feedback may help us to understand how we can improve the way we run the study if we continue working with other care homes. We do not anticipate there will be any risk in taking part in the interviews.

7. What if there is a problem?
If you have any worries about this project you should speak to the researcher or the lead for this research in your care home. If you remain concerned you can contact the PALS service (Patient Advice and Liaison Service, Bradford Royal Infirmary, Tel: 01274 364021).

8. Questions
If you have any questions or would like more information, please ask the Researcher directly.

If you would like further information about research in general, the UK Clinical Research Collaboration (a partnership of organisations working together on research in the UK) have published a booklet entitled 'Understanding Clinical Trials'. Contact UKCRC: Tel: 0207 670 5452; website www.ukcrc.org.

This project was funded by the National Institute for Health Research PGfAR (project number RP-RG-1210-12017)

Thank you for taking the time to read this information sheet.

This project was funded by the National Institute for Health Research PGfAR (project number RP-RG-1210-12017)

To be completed by REACH Researcher

Staff ID: _ _ _ _ _ _ Initials: _ _ _ _ DOB: _ _/_ _/_ _ _ _

Name of Care Home:……………………………………………………………………

The REACH Study

Research **E**xploring physical **A**ctivity in **C**are **H**omes

Staff Consent Form - Interviews

Please read the statements below and sign at the bottom if you agree to take part.

1.	I confirm that I have read the information sheet dated………………….. (Version ……….) and have had the chance to ask questions.
2.	I understand that taking part in the research interviews is my choice and that I am free to withdraw at any time without my employment being affected.
3.	I understand that even if I withdraw from the study, the data collected from me up to that point will be used in analysing the results of the study.
4.	I understand that the interview sessions will be recorded, transferred and stored securely at the Bradford Institute of Health Research.
5.	I agree for my details and a copy of this consent form (which will include my name and date of birth) to be stored by the Research Office (at the Bradford Institute of Health Research) for the purposes of this study.
6.	I agree to take part in the study.

This project was funded by the National Institute for Health Research PGfAR (project number RP-RG-1210-12017)

Staff member

Signature …………………………………………………………….

Name (block capitals) …………………………………….

Date ………………………….

Researcher

I have explained the study to the above named participant and he/she has indicated his/her willingness to participate.

Signature …………………………………………………………….

Name (block capitals) ……………………………………………….

Date ………………………….

(Original to be sent to BIHR; 1 copy for participant)

This project was funded by the National Institute for Health Research PGfAR (project number RP-RG-1210-12017)

To be completed by REACH Researcher

Name of Care Home:………………………………………………………………….

Date of workshop:……………………………….

No.of attendees:…………………….

The REACH Study

<u>R</u>esearch <u>E</u>xploring physical <u>A</u>ctivity in <u>C</u>are <u>H</u>omes

<u>Audio Recording of MoveMore Workshops - Consent Form</u>

Please ensure <u>ALL</u> the below points are covered at every workshop. Agreement to the below should be documented overleaf for every attendee.

- Your (or your relative/friends) Care Home is involved in the REACH study.
- We are asking you all to sign this form to be sure that you are happy for this MoveMore workshop to be audio recorded (by a member of the research team).
- Recordings will be reviewed by the research team so that they can find out what happened in each workshop.
- We would not keep any written records of your full name, although your name may be used during the workshops and so would be included in the recordings.
- However, any reports produced would be completely anonymous, which means that no-one other than the research team would know who had been part of the workshops.
- All recordings will be held securely by the Research Team at the Bradford Institute for Health Research until at least 5 years after the end of the study, and then confidentially destroyed.
- Sessions don't have to be recorded if anyone would rather this didn't happen.
- You can withdraw your consent for the recordings to be kept at any time prior to the study being analysed.

This project was funded by the National Institute for Health Research PGfAR (project number RP-RG-1210-12017)

To be completed by REACH Researcher

Name of Care Home:………………………………………………………………….

Date of workshop:………………………………….

No.of attendees:…………………….

Are all the attendees happy for the workshop sessions to be audio recorded?
Yes/No

Please note: Recordings can only be made if ALL workshop attendees agree.

Signature

……………………………………………

Name (block capitals)

……………………………………………

Date ………………………….

Signature

……………………………………………

Name (block capitals)

……………………………………………

Date ………………………….

Signature

……………………………………………

Name (block capitals)

……………………………………………

Date ………………………….

Signature

……………………………………………

Name (block capitals)

……………………………………………

Date …………………………….

Signature

……………………………………

Name (block capitals)

……………………………………

Date ……………………….

Signature

……………………………………………

Name (block capitals)

……………………………………………

Date ………………………….

Lead Researcher

Signature ………………………………………………………….

Name (block capitals) ……………………………………

Date ………………………….

(Original to be sent to the Research Office, 1 copy for participant)

This project was funded by the National Institute for Health Research PGfAR (project number RP-RG-1210-12017)
