# Supplementary material for: Research Exploring Physical Activity in Care Homes (REACH): study protocol for a randomised controlled trial
Source: Trials. 2017 Apr 19;18:182. doi: 10.1186/s13063-017-1921-8 (PMC5395795; doi:10.1186/s13063-017-1921-8)
Supplement: Supplementary file 3 — Progression criteria for continuation to the definitive randomised controlled trial. This document details guidelines for progression to a definitive randomised controlled trial (RCT) which are based on a traffic light system of green (proceed to RCT design), amber (review RCT design and/or implementation, then proceed), or red (stop and do not proceed). (DOCX 20 kb) [file 13063_2017_1921_MOESM3_ESM.docx]

**Progression criteria for continuation to the definitive randomised controlled trial**

Guidelines for progression to a definitive Randomised Controlled Trial (RCT) are based on a traffic light system of green (proceed to RCT design), amber (review RCT design and/or implementation, then proceed), red (stop and do not proceed), and are defined as follows:

**Green**

1. *Recruitment*

12 care homes are recruited; and
At least 20% of screened residents are eligible and consent to take part in the trial; and
On average each care home recruits at least 10 residents to the trial.

1. *Intervention delivery*

At least 75% of intervention care homes complete the series of three workshops and complete at least one observation review and one action plan review.

1. *Data collection & follow-up*

At least 75% of residents provide usable accelerometer data; and
At least 75% of residents have patient reported outcome measures from either themselves or a proxy; and
Loss to follow-up at 9 months is no greater than 25%; and
There are no safety concerns in the view of the Programme Steering Committee (PSC).

**Amber**

1. *Recruitment*

10 care homes are recruited; and
At least 10% of screened residents are eligible and consent to take part in the trial; and
On average each care home recruits at least 8 residents to the trial.

1. *Intervention delivery*

At least 50% of intervention care homes complete the series of three workshops and complete at least one observation review and one action plan review.

1. *Data collection & follow-up*

At least 65% of residents have patient reported outcome measures and;
Loss to follow-up at 9 months is no greater than 35% and;
There are no safety concerns in the view of the PSC.

**Red**

1. *Recruitment*

Less than 10 care homes are recruited; or
Less than 10% of screened residents are eligible and consent to take part in the trial; or
On average each care home recruits less than 8 residents to the trial

1. *Intervention delivery*

Less than 50% of intervention care homes complete the series of three workshops or less than 50% complete at least one observation review and one action plan review.

1. *Data collection & follow-up*

Less than 65% of residents have patient reported outcome measures; or
Loss to follow-up at 9 months is greater than 35%; or
There are major safety concerns in the view of the PSC.
